# Effects of glucocerebrosidase gene variations on the risk of Parkinson’s disease dementia: a meta-analysis

**DOI:** 10.3389/fnagi.2025.1671760

**Published:** 2025-11-14

**Authors:** Qiujie Li, Zhumei Bi, Weiming Liang, Shan Yin, Huaicheng Li, Zhongyou Liang, Minyao Wu, Jieru Quan, Cheng Li

**Affiliations:** 1 The First Affiliated Hospital of Guangxi University of Science and Technology, Guangxi University of Science and Technology, Liuzhou, Guangxi, China; 2 School of Economics and Management, Guangxi University of Science and Technology, Liuzhou, Guangxi, China; 3 Department of Geriatrics, Hezhou Guangji Hospital, Hospital, Hezhou, China

**Keywords:** glucocerebrosidase, Parkinson’s disease, dementia, meta-analysis, gene variations

## Abstract

**Objective:**

This meta-analysis aimed to investigate the effects of glucocerebrosidase gene (GBA) variations on the risk of Parkinson’s disease dementia (PDD) and to identify the relationship between GBA variations and PDD.

**Method:**

A comprehensive search was performed to retrieve publications from PubMed, Cochrane Library, Embase and Web of Science up to March 19, 2025. The search terms included “glucocerebrosidase,” “Parkinson’s disease,” and “dementia.” After rigorous screening, cohort studies were included for meta-analysis.

**Results:**

This meta-analysis revealed a significant overall association between the presence of GBA variation and an increased risk of dementia in PD patients (RR = 1.82, 95% CI: 1.52–2.18, *p* < 0.00001). When stratified by variant type, carriers of GBA mutations exhibited a similar elevation in dementia risk (RR = 1.82, 95% CI: 1.49–2.23, *p* < 0.00001), and carriers of GBA polymorphisms also demonstrated a heightened risk (RR = 1.82, 95% CI: 1.26–2.61, *p* = 0.001). Analysis of specific mutations revealed that the N370S variant was associated with an increase in dementia risk (RR = 1.54, 95% CI: 1.24–1.92, *p* < 0.0001), whereas the L444P variant conferred a stronger effect (RR = 2.17, 95% CI: 1.74–2.71, *p* < 0.00001). Additionally, the E326K polymorphism was also significantly associated with an increased risk of dementia (RR = 2.34, 95% CI: 1.88–2.91, *p* < 0.00001).

**Conclusion:**

GBA variations are significant risk factors for PDD, with varying degrees of risk conferred by different variants. These findings underscore the critical role of GBA in the pathogenesis of PDD and highlight its potential as a key genetic risk factor.

**Systematic review registration:**

https://www.crd.york.ac.uk/prospero/display_record.php?, Identifier CRD420251109378.

## 1 Introduction

Parkinson’s disease (PD) is the second most prevalent neurodegenerative disorder, affecting millions globally. It is primarily characterized by motor symptoms such as bradykinesia, rigidity, tremor, and postural instability, resulting from the progressive loss of dopaminergic neurons in the substantia nigra pars compacta (Kalia and Lang, 2015). However, PD is increasingly recognized as a complex disorder presenting a wide range of non-motor symptoms, including cognitive impairment, sleep disturbances, autonomic dysfunction, and psychiatric symptoms (Malec-Litwinowicz et al., 2014). Among these, Parkinson’s disease dementia (PDD) stands as one of the most debilitating non-motor complications, significantly impacting the quality of life for both patients and their caregivers, and contributing to increased morbidity and mortality (Aarsland and Kurz, 2010). PDD is defined by a decline in cognitive function, particularly in executive function, attention, and visuospatial skills, occurring within the context of established PD (Emre et al., 2007). The prevalence of PDD escalates with disease duration, affecting up to 80% of PD patients over the course of their illness (Hely et al., 2008). Dementia in Parkinson’s disease carries substantial adverse implications for quality of life, caregiver burden, and healthcare-related costs (Vossius et al., 2011).

Genetic factors play a pivotal role in the aetiology and progression of PD. While most PD cases are sporadic, a significant proportion, especially early-onset forms, have a genetic basis (Cacabelos, 2017). Over the past two decades, numerous genes have been identified as being associated with an elevated risk of PD, including SNCA, LRRK2, PARK7, PINK1, and GBA (Blauwendraat et al., 2020). These genetic discoveries have provided invaluable insights into the molecular pathways underpinning PD pathogenesis, such as alpha-synuclein aggregation, mitochondrial dysfunction, and lysosomal impairment (Schapira and Tolosa, 2010). Emerging evidence further suggests that certain genetic variations not only predispose individuals to PD but also influence the clinical phenotype and disease progression, including the development of cognitive decline and dementia (Lill, 2016; Agosta et al., 2013).

Deleterious mutations of GBA are defined as those associated with the onset of Gaucher disease and causative of PD in a heterozygous state, encompassing the common p.N370S and p.L444P (Beutler et al., 2005; Lesage et al., 2011). Sequence variants of exons with no identified relationships with PD in a heterozygous state are defined as GBA polymorphisms, including E326K, T369M, and E388K (Horowitz et al., 2011; Pankratz et al., 2012). GBA variations comprise the abovementioned GBA mutations and polymorphisms (Winder-Rhodes et al., 2013). These mutations are also considered an important risk factor for PD.

The glucocerebrosidase (GBA) gene, located on chromosome 1q21, encodes the lysosomal enzyme glucocerebrosidase (GCase). Mutations in GBA are well-established as the genetic cause of Gaucher disease, a lysosomal storage disorder (Rosenbloom and Weinreb, 2013). Crucially, GBA mutations are also recognized as the most common genetic risk factor for PD, with carriers exhibiting a significantly increased risk of developing the disease compared to non-carriers (Ye et al., 2023; Alcalay et al., 2012). Beyond its role in PD susceptibility, a growing body of research indicates that GBA variations are also strongly associated with an increased risk of developing PDD (Oftedal et al., 2023). The proposed mechanism involves reduced GCase activity, leading to the accumulation of its substrate, glucosylceramide, and subsequent lysosomal dysfunction. This, in turn, is thought to promote the aggregation and spread of alpha-synuclein, a hallmark pathological feature of PD and PDD (Jellinger, 2018).

Despite the accumulating evidence, studies investigating the association between GBA gene polymorphisms and mutations and the risk of dementia in PD patients have reported inconsistent findings. These discrepancies may arise from several factors, including differences in study populations, sample sizes, methodologies for assessing cognitive function, and the specific GBA variants analyzed (Riboldi and Di Fonzo, 2019). Some studies have identified a strong association between GBA mutations and PDD, while others have reported weaker or no significant links, particularly for certain polymorphisms or mild mutations (Gan-Or et al., 2018; Filippi et al., 2022). Given the clinical significance of identifying risk factors for PDD and the potential implications for personalized medicine, a comprehensive and systematic evaluation of the existing literature is warranted.

## 2 Materials and methods

### 2.1 Data sources and search strategy

This meta-analysis was conducted in accordance with the Preferred Reporting Items for Systematic Reviews and Meta-Analyses (PRISMA) guidelines (Moher et al., 2009). The study has been registered at PROSPERO with the registration number CRD420251109378. A comprehensive search was performed across major electronic databases, including PubMed, EMBASE, Web of Science, and the Cochrane Library, from their inception up to March 19, 2025. The search strategy was developed using a combination of Medical Subject Headings (MeSH) terms and free-text keywords related to Parkinson’s disease, glucocerebrosidase, and dementia. The search technique adhered to the PICOS principle and utilized a blend of MeSH terms and unrestricted text phrases. The search strategy employed combined the terms “Parkinson’s disease,” “glucocerebrosidase” and “dementia.” No language restrictions were applied during the initial search. Additionally, the reference lists of identified relevant articles and review papers were manually screened to identify any additional eligible studies.

### 2.2 Inclusion and exclusion criteria

Inclusion criteria: (1) patients diagnosed with Parkinson’s disease according to established diagnostic criteria, such as UK Parkinson’s Disease Society Brain Bank (UKPDSBB) Criteria (Hughes et al., 1992); (2) Exposure: Patients with genetically confirmed GBA variations, including but not limited to common mutations such as N370S and L444P, and polymorphisms like E326K; (3) Outcome: The incidence of dementia in GBA variant carriers compared to non-carriers within the PD patient cohort; (4) Types of study: Cohort studies.

Exclusion criteria were: (1) Not relevant; (2) other types of articles, such as conference abstracts yearbook, case reports, publications, letters, meta-analyses, reviews, retrospective studies, pharmacological intervention, animal studies and protocols; (3) Full text unavailable; (4) Data duplication; (5) Data could not be extracted for meta-analysis; (6) Case-control study designs.

### 2.3 Selection of studies

Study selection and duplicate removal were conducted using EndNote (Version 20; Clarivate Analytics). Two independent reviewers performed the initial screening by removing duplicate records, evaluating titles and abstracts for relevance, and categorising each study as either included or excluded. Disagreements were resolved through discussion and consensus. In cases where consensus could not be reached, a third reviewer served as an arbitrator to make the final decision.

### 2.4 Data extraction

Data were extracted by two reviewers independently. The extracted data included: (1) Basic study information, including the first author, publication year, country, study design, sample size, and main outcomes; (2) Baseline characteristics of study subjects, including number of patients, male ratio of patients, age at onset, disease duration, GBA genotype, and groups; (3) The data analyzed included total carriers and dementia cases for each GBA variations including GBA polymorphisms, GBA mutations and specific subtypes N370S, L444P, E326K, alongside equivalent data for non-GBA variant carriers. For studies reporting multiple GBA variants, data for each variant was extracted separately where possible. In the absence of consensus between the two independent reviewers, a third reviewer assumed the position of a mediator.

### 2.5 Quality assessment

The methodological quality of the included observational cohort studies was assessed using the Newcastle–Ottawa Scale (NOS) (Stang, 2010). The NOS evaluates studies based on three broad perspectives: selection of the study groups, comparability of the groups, and ascertainment of either the exposure or outcome of interest. A study can be awarded a maximum of nine stars, with higher scores indicating better methodological quality. Studies with a score of 7 or higher were considered to be of high quality, 4–6 of moderate quality, and less than 4 of low quality.

### 2.6 Statistical analysis

All statistical analyses were performed using Review Manager (RevMan) software and Stata12.0 software. The primary outcome measure was the risk ratio (RR) and its corresponding 95% confidence interval (CI) for the association between GBA variations and the risk of dementia in PD patients.

Due to the anticipated clinical and methodological heterogeneity among the included studies, a random-effects model was employed for all meta-analyses, which accounts for both within-study and between-study variability. Heterogeneity across studies was assessed using Cochran’s Q test and quantified by the *I*^2^ statistic (Cumpston et al., 2022). An *I*^2^ value of 0 to 40% was considered to represent unimportant heterogeneity, 30 to 60% moderate heterogeneity, 50 to 90% substantial heterogeneity, and 75 to 100% considerable heterogeneity (Higgins et al., 2003). A *p*-value <0.10 for the *Q* test or an *I*^2^ > 50% indicated significant heterogeneity, in which case the random-effects model was retained. If *I*^2^ was <50%, a fixed-effects model would have been considered.

Analyses were conducted for: overall GBA variations, GBA gene mutations (including N370S and L444P), and GBA gene polymorphisms (including E326K). Publication bias was visually inspected using funnel plots for outcomes. Sensitivity analyses, were conducted to evaluate the robustness of the pooled estimates by sequentially removing one study at a time and re-calculating the overall effect size. To quantitatively assess publication bias, Egger’s regression test was performed for each outcome, with a *p*-value <0.05 indicating significant publication bias. Furthermore, to address potential sources of heterogeneity and provide more detailed insights, subgroup analyses were performed based on ethnicity (e.g., Asian, Caucasian, Oceanian) for overall GBA variations and GBA mutations. Second, subgroup analyses were conducted based on dementia diagnostic criteria (e.g., DSM-IV, MDS, MMSE, CDR, MoCA) for overall GBA variations, GBA mutations, GBA polymorphisms, and specific variants (N370S, L444P, E326K).

## 3 Results

### 3.1 Search results

**Figure 1:**
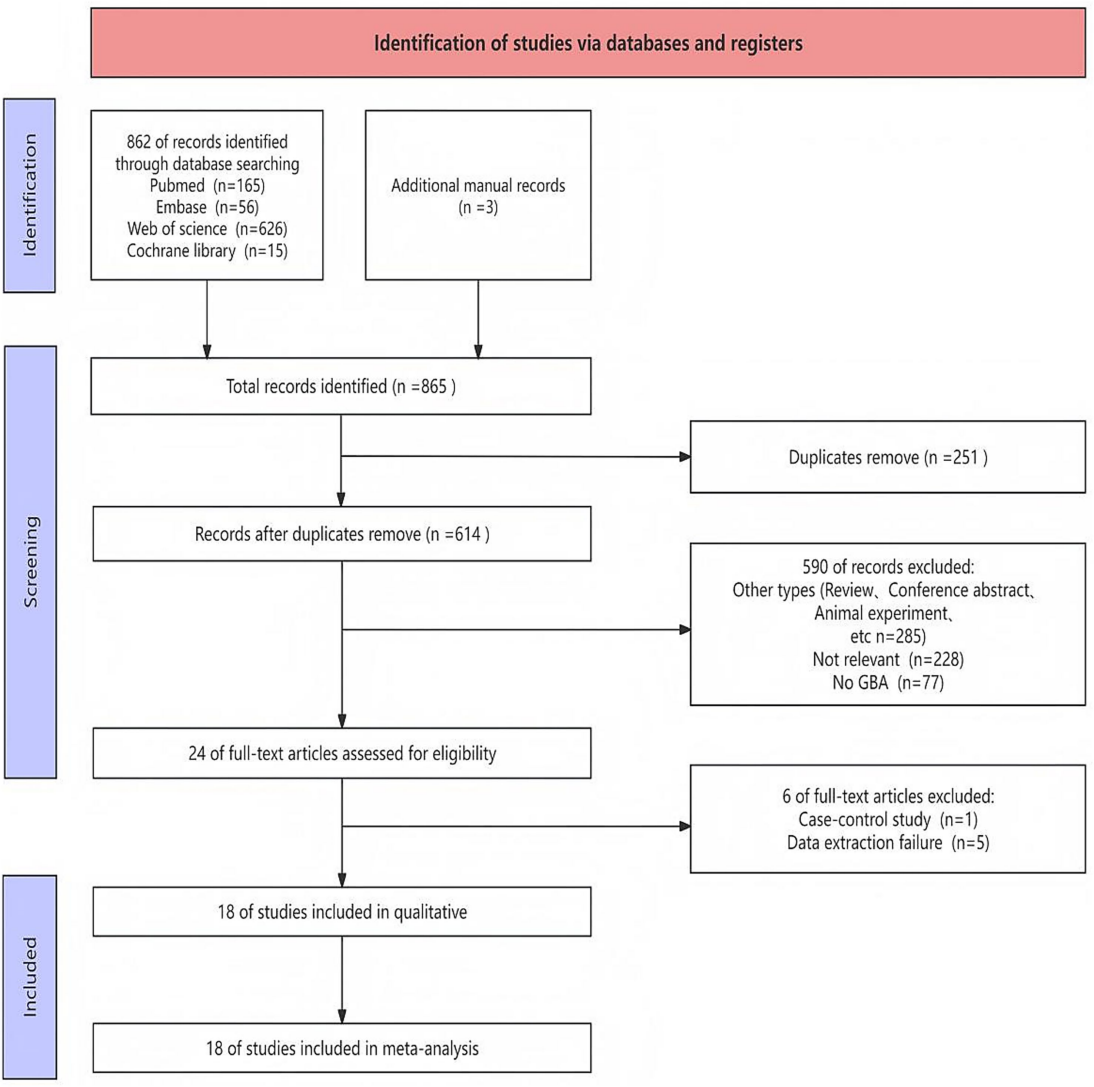
Flow chart of literature search strategies.

A comprehensive search was performed to retrieve publications regarding the effects of GBA on PDD risk from PubMed, Cochrane Library, Embase, and Web of Science. A total of 865 records were identified through database searching and additional manual records. After removal of duplicates, 614 unique records were screened based on their titles and abstracts. Of these, 24 full-text articles were retrieved for detailed assessment. After a comprehensive inspection of the entire text, a total of 18 article (Malec-Litwinowicz et al., 2014; Agosta et al., 2013; Alcalay et al., 2012; Chen et al., 2023; Cilia et al., 2016; Davis et al., 2016; De Michele et al., 2023; Graham et al., 2020; Lunde et al., 2018; Malek et al., 2018; Mata et al., 2016; Moran et al., 2017; Oeda et al., 2015; Setó-Salvia et al., 2012; Simuni et al., 2020; Straniero et al., 2020; Szwedo et al., 2022; Yahalom et al., 2019) were chosen for inclusion in this meta-analysis. The detailed study selection process was illustrated in the flow diagram (Figure 1), which outlined the number of records identified, screened, and included at each stage of the review.

### 3.2 Basic characteristics and quality assessment

The studies were published between 2012 and 2023, and originated from various countries, including USA, UK, Norway, Italy, Israel, Japan, China, Poland, Spain, and New Zealand. A total of 13,175 patients were included. Eighteen studies investigated the effects of GBA variations on PDD risk, 16 addressed the effects of GBA mutations on PDD risk, five explored the effects of GBA polymorphisms on PDD risk, four investigated the effects of GBA p.L444P on PDD risk, four explored the effects of GBA p.N370S on PDD risk, and three studied the effects of GBA p.E326K on PDD risk. The diagnostic criteria for PD and PDD varied across studies but were generally consistent with established clinical guidelines. The quality assessment using the Newcastle–Ottawa Scale (NOS) revealed that all 18 studies were of high quality (NOS score ≥7) (Table 1). A summary of the characteristics of the included studies is presented in Table 2.

**Table 1:** Quality assessment according to the NOS scale.

Author, year	Selection				Comparability		Outcome			Total scores
	Representativeness	Selection of non-exposure	Ascertainment of exposure	Outcome not present at start	Comparability on most important factors	Comparability on other risk factors	Assessment of outcome	Adequate follow-up time	Complete follow-up	
Agosta et al. (2013)	*	*	—	*	*	*	*	—	*	7
Alcalay et al. (2012)	*	—	*	*	*	*	*	—	*	7
Chen et al. (2023)	*	*	—	*	*	*	*	—	*	7
Cilia et al. (2016)	*	*	—	*	*	*	*	*	*	8
Davis et al. (2016)	*	—	*	*	*	—	*	*	*	7
De Michele et al. (2023)	*	*	—	*	*	*	*	*	*	8
Graham et al. (2020)	*	*	—	*	*	*	*	*	*	8
Lunde et al. (2018)	*	*	*	*	*	*	*	—	*	8
Malec-Litwinowicz et al. (2014)	*	—	*	*	*	*	*	*	—	7
Malek et al. (2018)	*	*	*	*	*	*	*	*	—	8
Mata et al. (2016)	*	*	*	*	*	—	*	—	*	7
Moran et al. (2017)	*	*	—	*	*	*	*	—	*	7
Oeda et al. (2015)	*	—	*	*	*	*	*	*	—	7
Setó-Salvia et al. (2012)	*	*	*	*	*	*	*	—	*	8
Simuni et al. (2020)	*	—	*	*	*	—	*	*	*	7
Straniero et al. (2020)	*	*	—	*	*	*	*	*	*	8
Szwedo et al. (2022)	*	*	—	*	*	*	*	*	*	8
Yahalom et al. (2019)	*	—	*	*	*	—	*	*	*	7

NOS, Newcastle–Ottawa Scale; “*” indicates criterion met; “—” indicates significant of criterion not.

**Table 2:** Characteristics of included studies and patients.

Author, year	Country	PD diagnostic criteria	Dementia evaluation method	Study design	Group	Sample size	Male%	Age at onset (mean ± SD)	Disease duration (mean ± SD)	*GBA* genotype
Agosta et al. (2013)	Italy	UK Brain Bank criteria	MDS	Cohort study	A	15	60.0	54 ± 7	10 ± 6	GBA variation; GBA mutation
					B	14	57.1	53 ± 8	11 ± 6	No variation
Alcalay et al. (2012)	USA	UK Brain Bank criteria	CDR, MMSE	Cohort study	A	26	69.2	42.9 ± 5.2	15.4 ± 5.8	GBA variation; GBA mutation
					B	39	59.0	43.6 ± 4.9	14.7 ± 5.4	No variation
Chen et al. (2023)	China	UK Brain Bank criteria	MDS	Cohort study	A	36	NA	NA	NA	GBA variation; GBA mutation
					B	196	NA	NA	NA	No variation
Cilia et al. (2016)	Italy	UK Brain Bank criteria	DSM-IV, MMSE	Cohort study	A	123	56.1	52.4 ± 10.2	11.9 ± 6.3	GBA variation; GBA mutation; N370S; L444P
					B	1982	60.8	57.4 ± 10.6	12.0 ± 6.6	No variation
Davis et al. (2016)	USA	UK Brain Bank criteria	MDS	Cohort study	A	58	55.2	NA	8.4 ± 5.2	GBA variation; GBA mutation; GBA polymorphism; E326K
					B	675	70.4	NA	8.7 ± 6.1	No variation
De Michele et al. (2023)	Italy	UK Brain Bank criteria	MDS	Cohort study	A	11	36.4	NA	9.2 ± 4.8	GBA variation; GBA mutation; L444P
					B	22	36.4	NA	8.8 ± 4.5	No variation
Graham et al. (2020)	New Zealand	UK Brain Bank criteria	MDS	Cohort study	A	21	71	58.3 ± 9.2	15.2 ± 6.9	GBA variation
					B	208	67.3	60.7 ± 8.5	13.7 ± 5.8	No variation
Lunde et al. (2018)	Norway	UK Brain Bank criteria	MDS	Cohort study	A	53	64.2	64.98 ± 9.79	NA	GBA variation; GBA mutation; GBA polymorphism
					B	389	59.9	68.03 ± 9.63	NA	No variation
Malec-Litwinowicz et al. (2014)	Poland	UK Brain Bank criteria	MMSE	Cohort study	A	5	NA	57.2 ± 2.8	NA	GBA variation; GBA mutation; N370S
					B	117	NA	57.6 ± 10.9	NA	No variation
Malek et al. (2018)	UK	UK Brain Bank criteria	MDS	Cohort study	A	142	65.5	64.3 ± 10.1	1.3 ± 1.0	GBA variation; GBA mutation; GBA polymorphism
					B	1,584	65.4	66.2 ± 9.2	1.3 ± 0.9	No variation
Mata et al. (2016)	USA	UK Brain Bank criteria	MDS	Cohort study	A	95	NA	55.8 ± 10.7	NA	GBA variation; GBA mutation; GBA polymorphism; E326K
					B	945	NA	59.7 ± 10.5	NA	No variation
Moran et al. (2017)	USA	UK Brain Bank criteria	DSM-IV	Cohort study	A	28	39	NA	NA	GBA variation; GBA mutation
					B	708	35.7	NA	NA	No variation
Oeda et al. (2015)	Japan	UK Brain Bank criteria	DSM-IV	Cohort study	A	19	26.3	55.2 ± 9.9	6.9 ± 4.6	GBA variation; GBA mutation
					B	196	50.0	59.4 ± 11.5	7.6 ± 5.4	No variation
Setó-Salvia et al. (2012)	Spain	UK Brain Bank criteria	CDR, DSM-IV	Cohort study	A	22	27.3	54.2 ± 6.6	14.1 ± 6.5	GBA variation; GBA mutation
					B	203	56.7	56.5 ± 12.7	12.0 ± 6.7	No variation
Simuni et al. (2020)	USA	UK Brain Bank criteria	MDS	Cohort study	A	80	53.8	58.4 ± 10.7	3.1 ± 2.0	GBA variation; GBA mutation
					B	361	65.9	59.7 ± 9.9	2.6 ± 0.6	No variation
Straniero et al. (2020)	Italy	UK Brain Bank criteria	DSM-IV, MDS	Cohort study	A	248	54.44	54.35 ± 10.96	14.05 ± 7.29	GBA variation; GBA mutation; N370S; L444P; GBA polymorphism; E326K
					B	3,433	60.30	58.24 ± 10.78	13.55 ± 6.89	No variation
Szwedo et al. (2022)	Norway	UK Brain Bank criteria	DSM-IV, MDS	Cohort study	A	100	63.7	65.3 ± 9.4	NA	GBA variation; GBA mutation; N370S; L444P
					B	867	60.8	67.6 ± 9.9	NA	No variation
Yahalom et al. (2019)	Israel	UK Brain Bank criteria	MoCA, MMSE	Cohort study	A	76	60.3	58.6 ± 10.0	11.5 ± 7.0	GBA variation
					B	78	65.0	61.4 ± 11.7	11.3 ± 6.5	No variation

A: Parkinson’s disease patients with GBA variation. B: Parkinson’s disease patients without GBA variation. NA, not available; MDS, Movement Disorder Society criteria; CDR, Clinical Dementia Rating; MMSE, Mini-Mental State Examination; DSM-IV, Diagnostic and Statistical Manual of Mental Disorders, Fourth Edition; MoCA, Montreal Cognitive Assessment.

### 3.3 Clinical outcomes

The meta-analysis results for clinical outcomes were consolidated and shown in Table 3.

**Table 3:** The results of the meta-analysis.

Outcomes	No. of study	Patients	Heterogeneity		Risk ratio	95% CI of overall effect
			*I*^2^ (%)	*p*-value		
GBA variations	18	13,175	66.00	0.00	1.82	1.52–2.18
GBA mutations	16	12,453	61.00	0.00	1.82	1.49–2.23
GBA polymorphisms	5	7,308	76.00	0.00	1.82	1.26–2.61
N370S	4	1,445	21.00	0.00	1.54	1.24–1.92
L444P	4	1,405	14.00	0.00	2.17	1.74–2.71
E326K	3	5,192	0.00	0.00	2.34	1.88–2.91

#### 3.3.1 Overall association of GBA variations with dementia risk

**Figure 2:**
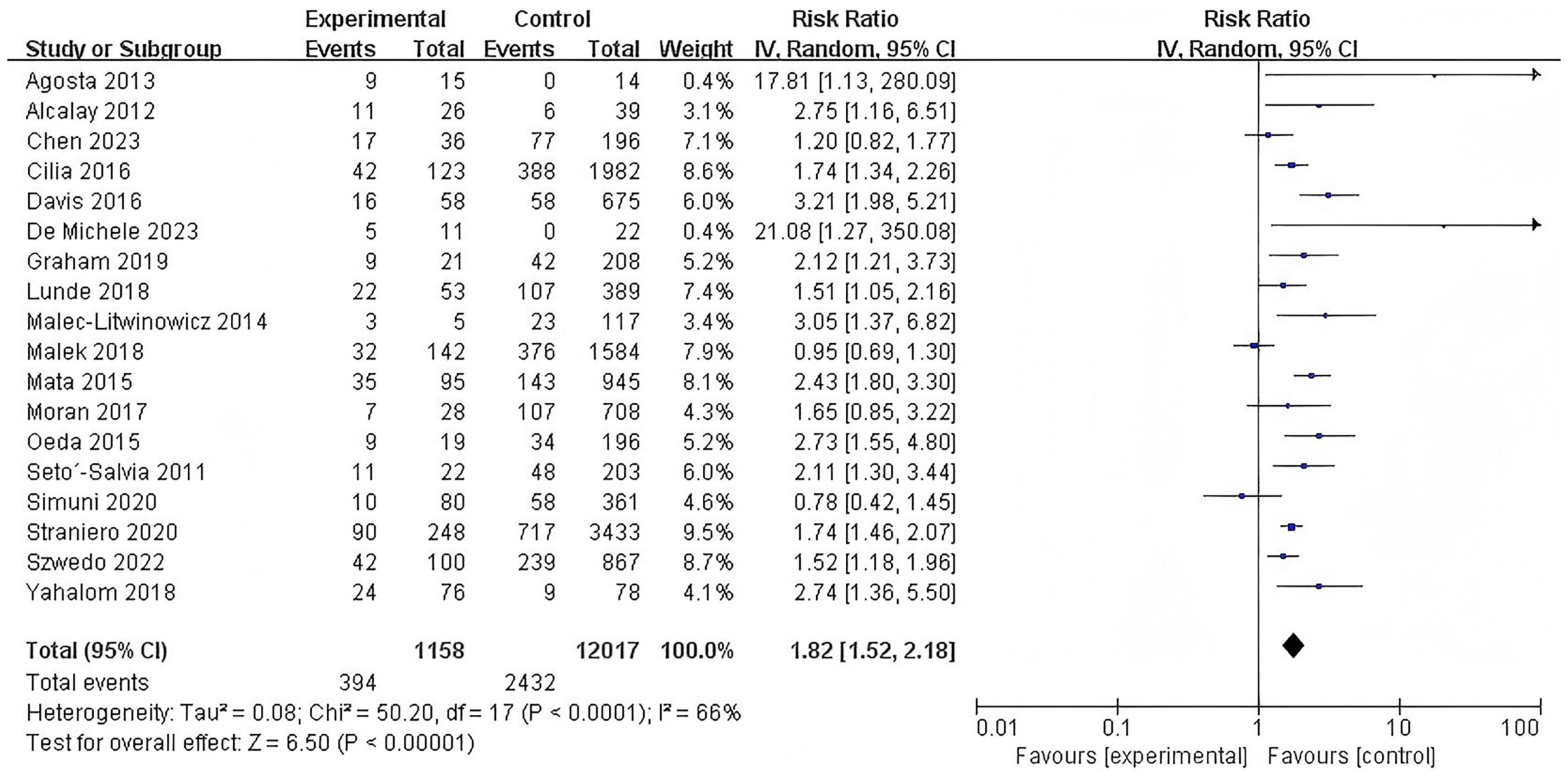
Forest plot of the meta-analysis for the overall association of GBA variations with dementia risk in PD patients.

This meta-analysis, which synthesizes data from 18 studies, revealed a significant overall association between the presence of GBA variations and an increased risk of dementia in Parkinson’s disease patients (RR = 1.82, 95% CI: 1.52–2.18, *p* < 0.00001, *I*^2^ = 66%), indicating that PD patients carrying GBA variations have an higher risk of developing dementia compared to non-carriers. Substantial heterogeneity was observed across these studies, justifying the use of a random-effects model (Figure 2).

#### 3.3.2 Association of GBA mutations with dementia risk

**Figure 3:**
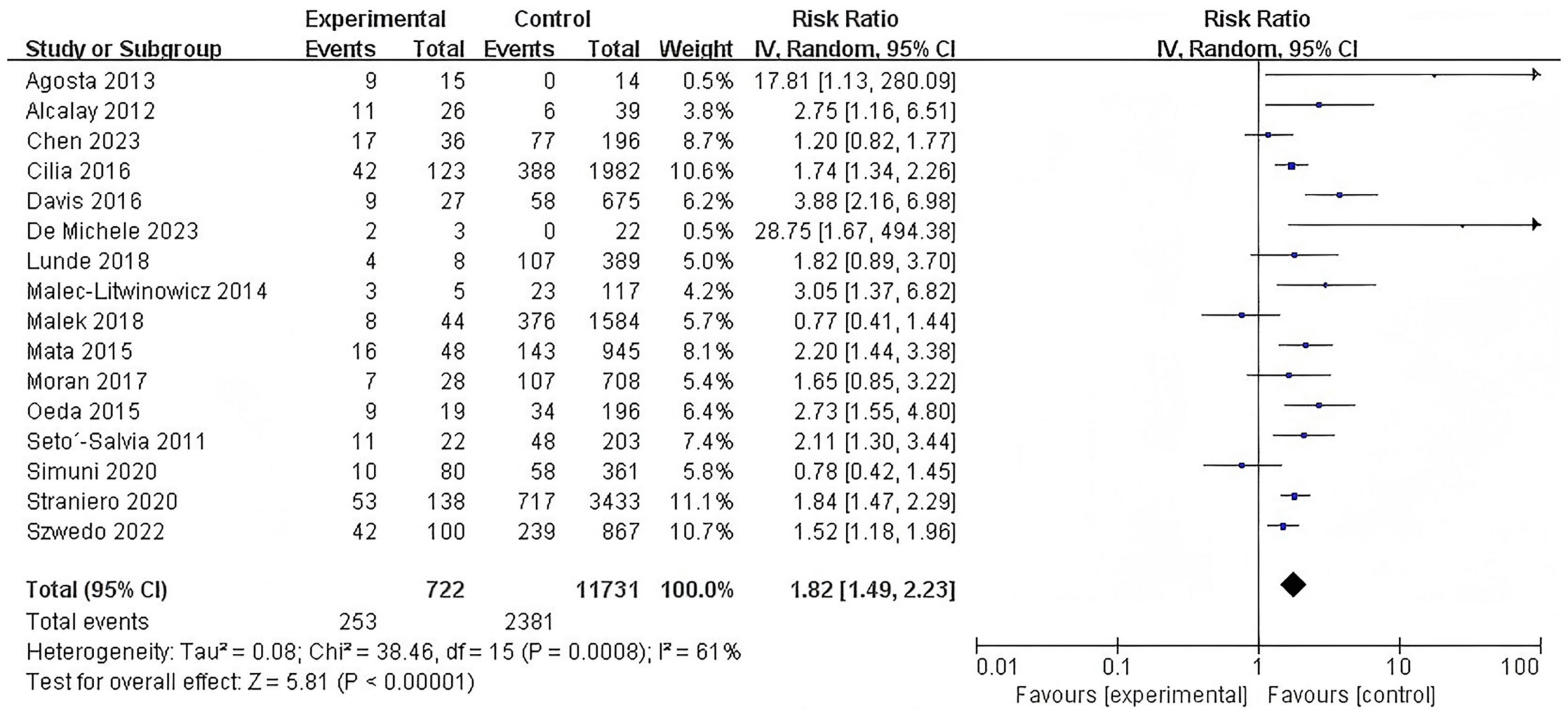
Forest plot of the meta-analysis for the association of GBA mutations with dementia risk in PD patients.

A subsequent subgroup analysis included 16 studies that investigated the association between GBA mutations and the risk of dementia in PD patients (RR = 1.82, 95% CI: 1.49–2.23, *p* < 0.00001, *I*^2^ = 61%), indicating that individuals with GBA mutations have a significantly increased risk of developing dementia. Heterogeneity was observed across these studies, suggesting some variability in effects among different mutation studies. This finding highlights the critical role of GBA mutations in the pathogenesis of Parkinson’s disease dementia (Figure 3).

#### 3.3.3 Association of GBA polymorphisms with dementia risk

**Figure 4:**
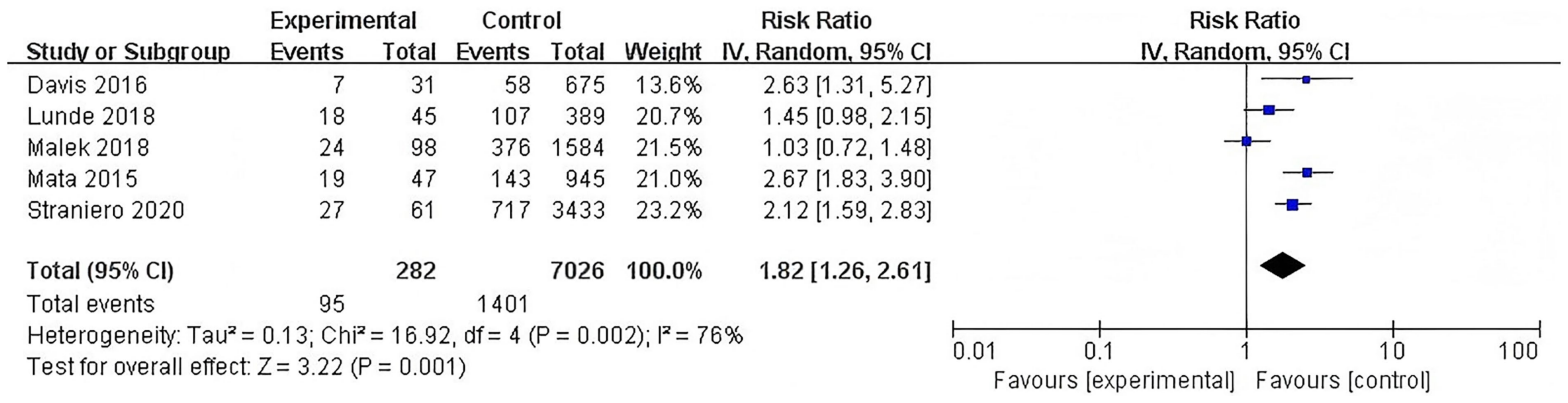
Forest plot of the meta-analysis for the association of GBA polymorphisms with dementia risk in PD patients.

Similarly, a separate subgroup analysis of studies examining GBA polymorphisms identified a significantly elevated risk of dementia among carriers (RR = 1.82, 95% CI: 1.26–2.61, *p* = 0.001, *I*^2^ = 76%). This finding underscores the important contribution of GBA polymorphisms to the genetic risk of PDD. However, there was substantial heterogeneity among the studies, suggesting considerable variability in the effect sizes across the included studies (Figure 4).

#### 3.3.4 Association of N370S mutation with dementia risk

**Figure 5:**
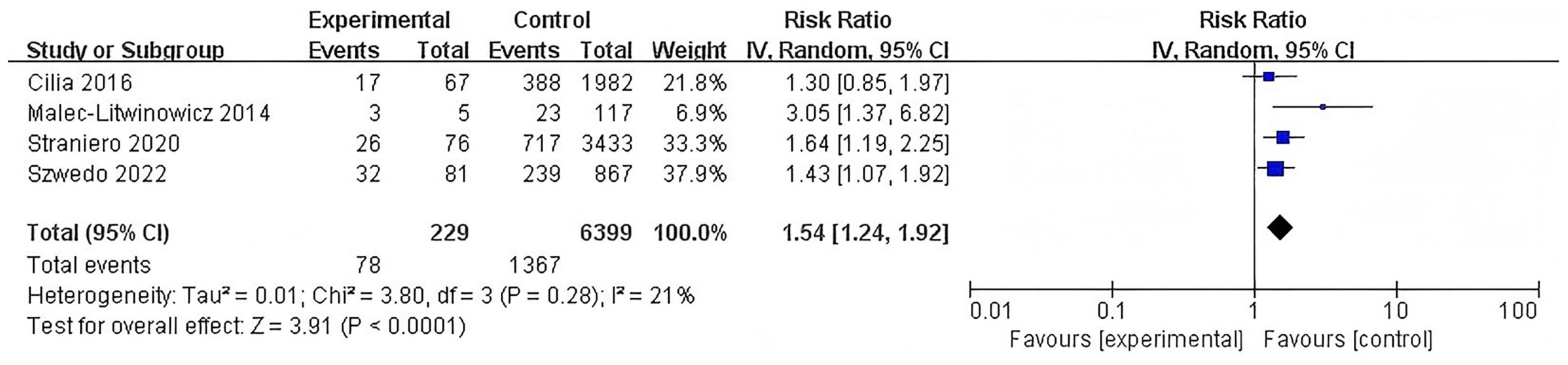
Forest plot of the meta-analysis for the association of N370S mutation with dementia risk in PD patients.

Further investigation into specific variants demonstrated that the N370S mutation is significantly associated with an increased risk of dementia, accompanied by low heterogeneity (RR = 1.54, 95% CI: 1.24–1.92, *p* < 0.0001, *I*^2^ = 21%) (Figure 5).

#### 3.3.5 Association of L444P mutation with dementia risk

**Figure 6:**
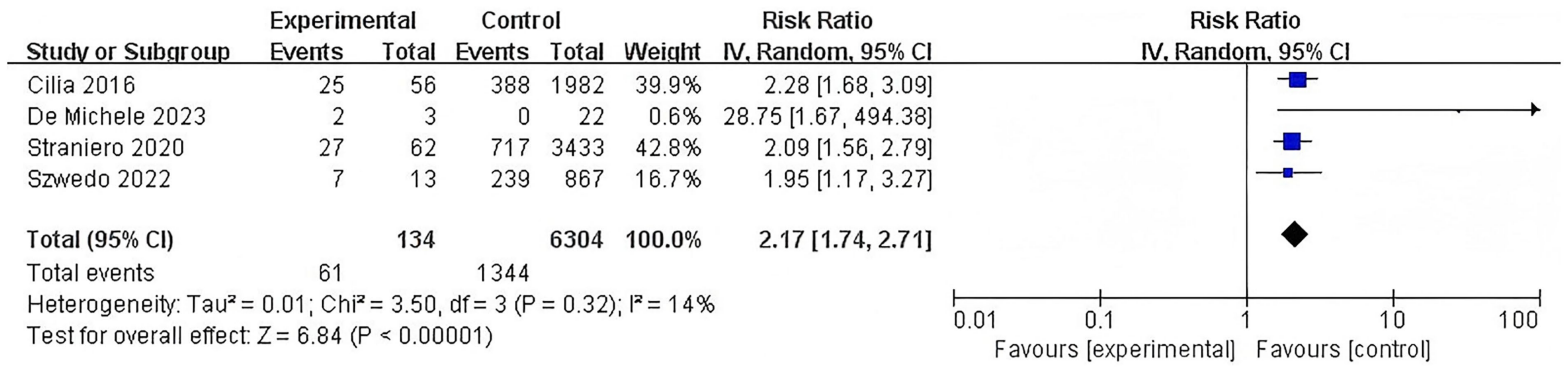
Forest plot of the meta-analysis for the association of L444P mutation with dementia risk in PD patients.

This meta-analysis determined that the L444P mutation was associated with a substantial increase in dementia risk associated with this severe GBA mutation and low heterogeneity was observed (RR = 2.17, 95% CI: 1.74–2.71, *p* < 0.00001, *I*^2^ = 14%) (Figure 6).

#### 3.3.6 Association of E326K polymorphism with dementia risk

**Figure 7:**
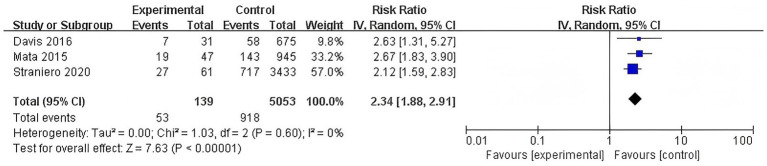
Forest plot of the meta-analysis for the association of E326K polymorphism with dementia risk in PD patients.

The E326K polymorphism was also significantly associated with an increased risk of dementia and no significant heterogeneity was detected for this subgroup (RR = 2.34, 95% CI: 1.88–2.91, *p* < 0.00001, *I*^2^ = 0%) (Figure 7).

### 3.4 Subgroup analyses

#### 3.4.1 Subgroup analysis by ethnicity

For GBA mutations, subgroup analysis by ethnicity revealed an RR of 1.76 (95% CI, 0.79–3.94, *p* = 0.17, *I*^2^ = 82%) for Asian populations and an RR of 1.86 (95% CI, 1.48–2.34, *p* < 0.00001, *I*^2^ = 63%) for Caucasian populations. The overall pooled RR for GBA mutations across all ethnicities was 1.83 (95% CI, 1.48–2.27, *p* < 0.00001, *I*^2^ = 64%). Crucially, the test for subgroup differences indicated that there was no significant heterogeneity between the ethnicity subgroups (Chi^2^ = 0.02, df = 1, *p* = 0.90, *I*^2^ = 0%), suggesting that ethnicity does not significantly modify the association between GBA mutations and PDD risk (Supplementary Figure 1).

For overall GBA variations, subgroup analysis by ethnicity showed an RR of 1.76 (95% CI: 0.79–3.94, *p* = 0.17, *I*^2^ = 82%) for Asian populations, an RR of 1.84 (95% CI: 1.49–2.27, *p* < 0.00001, *I*^2^ = 70%) for Caucasian populations, and an RR of 2.12 (95% CI: 1.21–3.73, *p* = 0.009) for Oceanian populations. The overall pooled RR for GBA variations across all ethnicities was 1.83 (95% CI: 1.52–2.21, *p* < 0.00001, *I*^2^ = 68%). The analysis for subgroup differences confirmed that ethnicity was not a significant source of heterogeneity (Chi^2^ = 0.24, df = 2, *p* = 0.89, *I*^2^ = 0%) (Supplementary Figure 2).

#### 3.4.2 Subgroup analysis by dementia diagnostic criteria

Subgroup analysis for GBA mutations based on dementia diagnostic criteria showed the following RRs: DSM-IV criteria (RR = 1.78, 95% CI: 1.56–2.02, *p* < 0.00001, *I*^2^ = 0%), MDS criteria (RR = 1.65, 95% CI: 1.23–2.23, *p* = 0.0010, *I*^2^ = 72%), CDR criteria (RR = 2.25, 95% CI: 1.48–3.44, *p* = 0.0002, *I*^2^ = 0%), and MMSE criteria (RR = 2.02, 95% CI: 1.44–2.82, *p* < 0.0001, *I*^2^ = 18%). The overall pooled RR for GBA mutations across all criteria was 1.80 (95% CI: 1.57–2.07, *p* < 0.00001, *I*^2^ = 52%). The test for subgroup differences revealed no significant impact of the diagnostic criteria used on the overall effect size (Chi^2^ = 1.87, df = 3, *p* = 0.60, *I*^2^ = 0%) (Supplementary Figure 3).

For overall GBA variations, subgroup analysis by dementia diagnostic criteria yielded these RRs: CDR criteria (RR = 2.25, 95% CI: 1.48–3.44, *p* = 0.0002, *I*^2^ = 0%), DSM-IV criteria (RR = 1.74, 95% CI: 1.55–1.96, *p* < 0.00001, *I*^2^ = 0%), MDS criteria (RR = 1.65, 95% CI: 1.29–2.11, *p* < 0.0001, *I*^2^ = 76%), MMSE criteria (RR = 2.07, 95% CI: 1.57–2.72, *p* < 0.00001, *I*^2^ = 12%), and MoCA criteria (RR = 2.74, 95% CI: 1.36–5.50, *p* = 0.005). The overall pooled RR for GBA variations across all criteria was 1.81 (95% CI: 1.59–2.06, *p* < 0.00001, *I*^2^ = 58%). Similarly, the test for subgroup differences indicated no significant influence of the diagnostic criteria on the risk estimate (Chi^2^ = 4.23, df = 4, *p* = 0.38, *I*^2^ = 5.3%) (Supplementary Figure 4).

For GBA polymorphisms, subgroup analysis by dementia diagnostic criteria showed an RR of 2.12 (95% CI: 1.59–2.83, *p* < 0.00001) for DSM-IV criteria and an RR of 1.82 (95% CI: 1.26–2.61, *p* = 0.001, *I*^2^ = 76%) for MDS criteria. The overall pooled RR was 1.86 (95% CI: 1.40–2.48, *p* < 0.0001, *I*^2^ = 72%). The test for subgroup differences was not significant (Chi^2^ = 0.43, df = 1, *p* = 0.51, *I*^2^ = 0%), suggesting consistent effects across the diagnostic criteria (Supplementary Figure 5).

For the N370S mutation, subgroup analysis by dementia diagnostic criteria showed an RR of 1.47 (95% CI: 1.22–1.78, *p* < 0.0001, *I*^2^ = 0%) for DSM-IV criteria, an RR of 1.52 (95% CI: 1.23–1.89, *p* = 0.0001, *I*^2^ = 0%) for MDS criteria, and an RR of 1.85 (95% CI: 0.81–4.24, *p* = 0.14, *I*^2^ = 71%) for MMSE criteria. The overall pooled RR was 1.50 (95% CI: 1.32–1.72, *p* < 0.00001, *I*^2^ = 0%). The test for subgroup differences indicated no significant heterogeneity across the different diagnostic criteria (Chi^2^ = 0.30, df = 2, *p* = 0.86, *I*^2^ = 0%) (Supplementary Figure 6).

For the L444P mutation, subgroup analysis by dementia diagnostic criteria showed an RR of 2.14 (95% CI: 1.76–2.60, *p* < 0.00001, *I*^2^ = 0%) for DSM-IV criteria, an RR of 2.16 (95% CI: 1.40–3.32, *p* = 0.0005, *I*^2^ = 40%) for MDS criteria, and an RR of 2.28 (95% CI: 1.68–3.09, *p* < 0.00001) for MMSE criteria. The overall pooled RR was 2.16 (95% CI: 1.88–2.47, *p* < 0.00001, *I*^2^ = 0%). The test for subgroup differences showed no significant impact of diagnostic criteria on the risk estimate (Chi^2^ = 0.12, df = 2, *p* = 0.94, *I*^2^ = 0%) (Supplementary Figure 7).

For the E326K polymorphism, subgroup analysis by dementia diagnostic criteria showed an RR of 2.12 (95% CI: 1.59–2.83, *p* < 0.00001) for DSM-IV criteria and an RR of 2.34 (95% CI: 1.88–2.91, *p* < 0.00001, *I*^2^ = 0%) for MDS criteria. The overall pooled RR was 2.26 (95% CI: 1.90–2.68, *p* < 0.00001, *I*^2^ = 0%). The test for subgroup differences was not significant (Chi^2^ = 0.28, df = 1, *p* = 0.60, *I*^2^ = 0%), indicating that the choice of diagnostic criteria did not introduce heterogeneity (Supplementary Figure 8).

### 3.5 Sensitivity analysis

To further evaluate the stability and robustness of the pooled estimates, sensitivity analysis was performed. This method involves systematically removing one study at a time from the meta-analysis and re-calculating the overall effect size. The purpose is to identify whether any single study disproportionately influences the overall pooled estimate, which could indicate a lack of robustness or the presence of an outlier study. The results of the sensitivity analysis for the overall association of GBA variations with dementia risk (Supplementary Figure 9), as well as for the GBA mutations (Supplementary Figure 10), GBA polymorphisms (Supplementary Figure 11), N370S mutation (Supplementary Figure 12), L444P mutation (Supplementary Figure 13), and E326K polymorphism (Supplementary Figure 14), consistently demonstrated that no single study had an undue influence on the respective pooled estimates. The recalculated effect sizes remained within a narrow range, and the statistical significance of the associations was maintained across all iterations. This consistency across the sensitivity analyses strongly confirms the stability and robustness of the findings, indicating that the conclusions are not driven by any single study and are reliable despite the observed heterogeneity.

### 3.6 Publication bias analysis

Publication bias was visually inspected using funnel plots for outcomes. For the overall association of GBA variations, GBA mutations and GBA polymorphisms, with dementia risk, the funnel plot (Supplementary Figures 15–17) suggested some asymmetry. This asymmetry could potentially indicate the presence of publication bias. The funnel plots of p.N370S, p.L444P and p.E326K are basically contralateral. Each score is scattered on both sides of the midline and is within the 95% CI, with no obvious missing angles. This suggests a small possibility in publication bias (Supplementary Figures 18–20).

To provide a quantitative assessment of publication bias, Egger’s regression test was performed. The results indicated no significant publication bias for N370S (*t* = 1.41, *p* = 0.293) (Supplementary Figure 21), L444P (*t* = 1.87, *p* = 0.203) (Supplementary Figure 22), E326K (*t* = 0.84, *p* = 0.553) (Supplementary Figure 23), GBA polymorphisms (*t* = 0.21, *p* = 0.850) (Supplementary Figure 24), GBA mutations (*t* = 1.36, *p* = 0.196) (Supplementary Figure 25), and overall GBA variations (*t* = 1.66, *p* = 0.117) (Supplementary Figure 26). These quantitative findings complement the visual inspection of funnel plots and further support the robustness of the meta-analysis results against publication bias.

## 4 Discussion

This meta-analysis provides compelling evidence that GBA gene variations, encompassing both mutations and polymorphisms, are significantly associated with an increased risk of dementia in patients with Parkinson’s disease. The findings demonstrate that PD patients carrying GBA variation have an approximately 82% higher risk of developing dementia compared to non-carriers. This robust association underscores the critical role of GBA in the pathogenesis of PDD and highlights its potential as a key genetic risk factor (Straniero et al., 2020). Furthermore, analyses revealed that both severe mutations (L444P), mild mutations (N370S), and even the common polymorphism (E326K) are independently associated with an elevated risk of PDD, with varying degrees of risk (Malec-Litwinowicz et al., 2014; De Michele et al., 2023; Mata et al., 2016; Setó-Salvia et al., 2012; Straniero et al., 2020). Notably, the L444P mutation showed the highest risk ratio, followed by the E326K polymorphism and the N370S mutation, suggesting a potential correlation between the severity of the GBA variant and the magnitude of dementia risk. This observation aligns with the understanding that different GBA variants may lead to varying degrees of GCase enzyme deficiency, thereby differentially impacting downstream pathological processes.

The comprehensive subgroup analyses performed in this study provide further insights into the influence of ethnicity and dementia diagnostic criteria on the observed associations. The consistent findings across different ethnic groups for GBA mutations and variations suggest a broad applicability of these genetic risk factors. Moreover, the varying risk ratios observed across different dementia diagnostic criteria highlight the importance of standardized diagnostic approaches in future research and clinical practice. These detailed subgroup analyses enhance the generalizability and clinical relevance of the findings, addressing potential sources of heterogeneity that could confound the overall estimates.

The underlying mechanisms linking GBA variations to PDD are complex and likely involve lysosomal dysfunction and altered alpha-synuclein homeostasis. The GBA gene encodes glucocerebrosidase, a lysosomal enzyme responsible for the hydrolysis of glucosylceramide to glucose and ceramide (Grabowski, 2012; Chatterjee and Krainc, 2023). Mutations in GBA lead to reduced GCase activity, resulting in the accumulation of its substrate within lysosomes (Oftedal et al., 2023). This lysosomal dysfunction is hypothesized to impair the clearance of alpha-synuclein, leading to its aggregation and the formation of Lewy bodies, which are neuropathological hallmarks of both PD and PDD (Lee et al., 2013; Do et al., 2019). The accumulation of misfolded alpha-synuclein can further exacerbate lysosomal dysfunction, creating a vicious cycle that contributes to neurodegeneration and cognitive decline (Cerri et al., 2018; Silva et al., 2025; Rocha et al., 2023; Smith and Schapira, 2022). Moreover, reduced GCase activity may also impact other cellular processes, including mitochondrial function, oxidative stress, and neuroinflammation, all of which are implicated in PD pathogenesis and PDD development (Smith and Schapira, 2022; Gegg and Schapira, 2018; Atashrazm et al., 2018). The varying risk levels observed for different GBA variants could be attributed to their differential impact on GCase activity and subsequent cellular consequences (Smith and Schapira, 2022). For instance, severe mutations like L444P may lead to a more profound reduction in GCase activity, resulting in a greater burden of alpha-synuclein pathology and a higher risk of dementia, compared to milder mutations or polymorphisms (Granek et al., 2023; Bendikov-Bar et al., 2011).

Recent advances in the understanding of PDD, particularly since 2020, have illuminated several interconnected mechanisms that extend beyond traditional models. Lysosomal dysfunction, directly linked to GBA mutations, remains a central theme. Impaired GCase activity leads to the accumulation of glycosphingolipids, which promotes α-synuclein aggregation and neuroinflammation (Calabresi et al., 2023). This has spurred the development of pharmacological chaperones and enzyme replacement therapies, which have shown promise in preclinical and early clinical settings (Pardo-Moreno et al., 2023). Mitochondrial dysfunction has also been identified as a critical factor. GBA mutations can indirectly impair mitochondrial function, leading to oxidative stress and contributing to neuronal damage. Consequently, therapeutic strategies targeting mitochondrial health, such as coenzyme Q10 supplementation and novel mitochondrial-targeted antioxidants, are under active investigation (Colca and Finck, 2022). Neuroinflammation, mediated by activated microglia and astrocytes, is increasingly recognized as a key driver of PDD progression. GBA mutations appear to exacerbate these neuroinflammatory responses. As such, immunomodulatory therapies targeting specific inflammatory pathways represent a promising new frontier in treatment development (Iarkov et al., 2021). Furthermore, the gut-brain axis has emerged as a significant area of research. Dysbiosis of the gut microbiota can influence neuroinflammation and α-synuclein pathology. Interventions such as probiotics, prebiotics, and faecal microbiota transplantation are being explored for their potential to modulate disease progression (Klann et al., 2021).

The findings of this meta-analysis have significant clinical implications. The identification of GBA gene variations as a strong risk factor for PDD suggests that genetic screening for GBA variants could be a valuable tool for assessing dementia risk in PD patients. Early identification of high-risk individuals could enable more targeted monitoring, earlier intervention strategies, and personalized management plans (Moore and Barker, 2014; Szwedo et al., 2025). For example, patients carrying GBA variants might benefit from more frequent cognitive assessments, or be prioritized for clinical trials investigating novel therapies aimed at improving lysosomal function or reducing alpha-synuclein aggregation (Zhang et al., 2019; Ciccaldo et al., 2025; Williams et al., 2024a; Williams et al., 2024b). Furthermore, understanding the specific GBA variants and their associated risk levels could help clinicians provide more accurate prognoses and counsel patients and their families more effectively regarding the potential trajectory of their disease (Menozzi et al., 2023). This personalized approach to care, informed by genetic insights, represents a significant step towards improving outcomes for PD patients at risk of developing dementia (Cook et al., 2021; Hill et al., 2022).

The clinical significance of these findings is substantial. The integration of GBA gene screening into routine clinical practice for PD patients could serve as a valuable tool for early risk assessment of dementia. This genetic information can help clinicians identify individuals at higher risk for PDD, enabling the implementation of more proactive and individualized management strategies, including targeted monitoring, early cognitive interventions, and personalized therapeutic approaches. Ultimately, understanding the genetic predisposition to PDD, particularly concerning GBA variations, holds promise for improving patient outcomes and advancing the development of precision medicine in Parkinson’s disease.

This meta-analysis builds upon previous research by providing a comprehensive and updated assessment of the association between GBA gene variations and the risk of Parkinson’s disease dementia. This study distinguishes itself from prior work, such as the 2020 meta-analysis by Zhang et al. (2020) which similarly concluded that GBA polymorphisms and mutations increase PDD risk.

Firstly, it represents the most comprehensive meta-analysis to date specifically focusing on the association between GBA gene polymorphisms and mutations and the risk of dementia in PD patients. By incorporating a larger and more recent dataset, it significantly enhances statistical power and the timeliness of the findings. Secondly, the systematic search strategy across multiple databases minimized the risk of publication bias, and the independent data extraction and quality assessment enhanced the reliability of the findings. This study quantitatively assesses publication bias using Egger’s regression test, complementing visual inspection of funnel plots to ensure the robustness of the pooled estimates. Thirdly, the use of a random-effects model appropriately accounted for the inherent heterogeneity across studies, providing a more conservative and generalizable estimate of the effect. Finally, the separate analyses for different GBA variants, coupled with refined methodological approaches such as detailed subgroup analyses based on ethnicity and various dementia diagnostic criteria, allowed for a detailed examination of their impact. This provides more granular insights into specific GBA variants and their differential effects on PDD risk, exploring potential sources of heterogeneity.

Furthermore, this meta-analysis acknowledges the existence of GBA variants beyond N370S, L444P, and E326K, including other Gaucher disease-related pathogenic mutations (e.g., D409H, R463C, RecNciI complex allele) and other polymorphisms (e.g., E388K, R120W, IVS10+1G>T), which may contribute to PD and PDD risk in specific populations. Future research should consider expanding genetic screening to include these less common but clinically relevant variants, potentially through whole-genome sequencing approaches. By addressing these aspects, this meta-analysis seeks to provide a more robust estimate of the association and to clarify whether different GBA variants confer varying degrees of risk for PDD, thereby offering valuable insights for clinical practice and future research directions.

Despite these strengths, this meta-analysis also has several limitations. The observed heterogeneity, particularly in the overall analysis, suggests that other factors not accounted for in the analysis may influence the association between GBA variations and PDD. These factors could include differences in patient demographics, disease duration, concomitant medications, and the specific diagnostic criteria used for PDD across studies. While analyses were performed for specific GBA variants, further stratification by other clinical or genetic factors was limited by the available data in the included studies. Additionally, the reliance on published data means that individual patient data could not be accessed, which would have allowed for more detailed analyses and adjustment for potential confounders. The visual inspection of funnel plots suggested some asymmetry, indicating a potential for publication bias. Although Egger’s regression tests did not indicate significant publication bias for individual variants and overall categories, this does not entirely rule out the possibility of bias, especially for subgroups with fewer studies. Future research should aim to address these limitations by conducting larger, prospective cohort studies with standardized methodologies for GBA genotyping, PDD diagnosis, and comprehensive collection of clinical and demographic data. Further mechanistic studies are also needed to fully elucidate the complex interplay between GBA dysfunction, alpha-synuclein pathology, and cognitive decline in PD (Moore and Barker, 2014).

In conclusion, this comprehensive meta-analysis provides robust evidence confirming that both mutations and polymorphisms in the GBA gene are significantly associated with an increased risk of dementia in patients with Parkinson’s disease. The findings highlight that different GBA variants confer varying degrees of risk, with severe mutations, mild mutations, and even common polymorphisms all contributing to an elevated likelihood of developing PDD. This study underscores the critical role of GBA in the genetic landscape of PDD and its potential as a predictive biomarker. The detailed subgroup analyses and quantitative assessment of publication bias further solidify these conclusions, offering a more refined understanding of GBA’s impact on PDD risk across diverse clinical and demographic contexts.

## Data Availability

The datasets presented in this study can be found in online repositories. The names of the repository/repositories and accession number(s) can be found in the article/Supplementary material.
