# Supplementary material for: Effects of glucocerebrosidase gene variations on the risk of Parkinson’s disease dementia: a meta-analysis
Source: Front Aging Neurosci. 2025 Nov 14;17:1671760. doi: 10.3389/fnagi.2025.1671760 (PMC12660284; doi:10.3389/fnagi.2025.1671760)
Supplement: Supplementary file 1 [file Data_Sheet_1.pdf]

The combination of MeSH and free-text words regarding the keywords

(1)Glucocerebrosidase

beta-Glucocerebrosidase
beta Glucocerebrosidase
Acid beta-Glucosidase
Acid beta Glucosidase
beta-Glucosidase, Acid
Glucocerebroside beta-Glucosidase
Glucocerebroside beta Glucosidase
beta-Glucosidase, Glucocerebroside
Glucosylceramide beta-Glucosidase
Glucosylceramide beta Glucosidase
beta-Glucosidase, Glucosylceramide
Glucosylsphingosine Glucosyl Hydrolase
Glucosyl Hydrolase, Glucosylsphingosine
Hydrolase, Glucosylsphingosine Glucosyl
Glucosyl Ceramidase
Ceramidase, Glucosyl
beta glucosylceramide glucosidase
d glucosyl n acylsphingosine glucohydrolase
e.c. 3.2.1.45
glucosylceramide glucosidase
recombinant human derived r-glucacerebrosidase (r-gcr)
glucosylceramidase

(2)Parkinson's Disease

Idiopathic Parkinson's Disease
Lewy Body Parkinson's Disease
Parkinson's Disease, Idiopathic
Parkinson's Disease, Lewy Body
Parkinson Disease, Idiopathic
Idiopathic Parkinson Disease
Lewy Body Parkinson Disease
Primary Parkinsonism
Parkinsonism, Primary
Paralysis Agitans
idiopathic parkinsonism
Lewy bodies of Parkinson disease
Lewy bodies of Parkinson`s disease
Lewy bodies of Parkinsons disease

Lewy body Parkinson disease
Lewy body Parkinson`s disease
Lewy body Parkinsons disease
paralysis agitans
Parkinson dementia complex
Parkinsons disease
primary parkinsonism
Parkinson disease

(3)dementia
Dementias
Amentia
Amentias
Senile Paranoid Dementia
Dementias, Senile Paranoid
Paranoid Dementia, Senile
Paranoid Dementias, Senile
Senile Paranoid Dementias
Familial Dementia
Dementia, Familial
Dementias, Familial
Familial Dementias
demention

Comprehensive listing of the search results.

PubMed		
No.	Query	Results
1	((((((((((((((((((((beta-Glucocerebrosidase[Title/Abstract]) OR (beta Glucocerebrosidase[Title/Abstract])) OR (Acid beta-Glucosidase[Title/Abstract])) OR (Acid beta Glucosidase[Title/Abstract])) OR (beta-Glucosidase, Acid[Title/Abstract])) OR (Glucocerebroside beta-Glucosidase[Title/Abstract])) OR (Glucocerebroside beta Glucosidase[Title/Abstract])) OR (beta-Glucosidase, Glucocerebroside[Title/Abstract])) OR (Glucosylceramide beta-Glucosidase[Title/Abstract])) OR (Glucosylceramide beta Glucosidase[Title/Abstract])) OR (beta-Glucosidase, Glucosylceramide[Title/Abstract])) OR (Glucosylsphingosine Glucosyl Hydrolase[Title/Abstract])) OR (Glucosyl Hydrolase, Glucosylsphingosine[Title/Abstract])) OR (Hydrolase, Glucosylsphingosine Glucosyl[Title/Abstract])) OR (Glucosyl Ceramidase[Title/Abstract])) OR (Ceramidase, Glucosyl[Title/Abstract])) OR (Glucocerebrosidase[Title/Abstract])) OR (beta glucosylceramide glucosidase[Title/Abstract])) OR (d glucosyl n acylsphingosine glucohydrolase[Title/Abstract])) OR (e.c. 3.2.1.45[Title/Abstract])) OR (glucosylceramide glucosidase[Title/Abstract])) OR (recombinant human derived r-glucacerebrosidase (r-gcr[Title/Abstract]))) OR (glucosylceramidase[Title/Abstract])	3146
2	((((((((((((((((((((Idiopathic Parkinson's Disease[Title/Abstract]) OR (Lewy Body Parkinson's Disease[Title/Abstract])) OR (Parkinson's Disease, Idiopathic[Title/Abstract])) OR (Parkinson's Disease, Lewy Body[Title/Abstract])) OR (Parkinson Disease, Idiopathic[Title/Abstract])) OR (Parkinson's Disease[Title/Abstract])) OR (Idiopathic Parkinson Disease[Title/Abstract])) OR (Lewy Body Parkinson Disease[Title/Abstract])) OR (Primary Parkinsonism[Title/Abstract])) OR (Parkinsonism, Primary[Title/Abstract])) OR (Paralysis Agitans[Title/Abstract])) OR (idiopathic parkinsonism[Title/Abstract])) OR (Lewy bodies of Parkinson disease[Title/Abstract])) OR (Lewy bodies of Parkinson`s disease[Title/Abstract])) OR (Lewy bodies of Parkinsons disease[Title/Abstract])) OR (Lewy body Parkinson disease[Title/Abstract])) OR (Lewy body Parkinson`s disease[Title/Abstract])) OR (Lewy body Parkinsons disease[Title/Abstract])) OR (paralysis agitans[Title/Abstract])) OR (Parkinson dementia complex[Title/Abstract])) OR (Parkinsons disease[Title/Abstract])) OR (primary parkinsonism[Title/Abstract]))	127366

	OR (Parkinson disease[Title/Abstract])	
3	((((((((((((Dementias[Title/Abstract]) OR (Amentia[Title/Abstract])) OR (Amentias[Title/Abstract])) OR (Senile Paranoid Dementia[Title/Abstract])) OR (Dementias, Senile Paranoid[Title/Abstract])) OR (Paranoid Dementia, Senile[Title/Abstract])) OR (Paranoid Dementias, Senile[Title/Abstract])) OR (Senile Paranoid Dementias[Title/Abstract])) OR (Familial Dementia[Title/Abstract])) OR (Dementia, Familial[Title/Abstract])) OR (Dementias, Familial[Title/Abstract])) OR (Familial Dementias[Title/Abstract])) OR (demention[Title/Abstract])) OR (dementia[Title/Abstract])	154232
4	1 AND 2 AND 3	165

Cochrane library		
No.	Query	Results
1	(beta-Glucocerebrosidase OR beta Glucocerebrosidase OR Acid beta-Glucosidase OR Acid beta Glucosidase OR beta-Glucosidase, Acid OR Glucocerebroside beta-Glucosidase OR Glucocerebroside beta Glucosidase OR beta-Glucosidase, Glucocerebroside OR Glucosylceramide beta-Glucosidase OR Glucosylceramide beta Glucosidase OR beta-Glucosidase, Glucosylceramide OR Glucosylsphingosine Glucosyl Hydrolase OR Glucosyl Hydrolase, Glucosylsphingosine OR Hydrolase, Glucosylsphingosine Glucosyl OR Glucosyl Ceramidase OR Ceramidase, Glucosyl OR Glucocerebrosidase OR beta glucosylceramide glucosidase OR d glucosyl n acylsphingosine glucohydrolase OR e.c. 3.2.1.45 OR glucosylceramide glucosidase OR recombinant human derived r-glucacerebrosidase (r-gcr) OR glucosylceramidase):ti,ab,kw	157
2	(Idiopathic Parkinson's Disease OR Lewy Body Parkinson's Disease OR Parkinson's Disease, Idiopathic OR Parkinson's Disease, Lewy Body OR Parkinson Disease, Idiopathic OR Parkinson's Disease OR Idiopathic Parkinson Disease OR Lewy Body Parkinson Disease OR Primary Parkinsonism OR Parkinsonism, Primary OR Paralysis Agitans OR idiopathic parkinsonism OR Lewy bodies of Parkinson disease OR Lewy bodies of Parkinson`s disease OR Lewy bodies of Parkinsons disease OR Lewy body Parkinson disease OR Lewy body Parkinson`s disease OR Lewy body Parkinsons disease OR paralysis agitans OR Parkinson dementia complex OR Parkinsons disease OR primary parkinsonism OR Parkinson disease):ti,ab,kw	13480
3	(Dementias OR Amentia OR Amentias OR Senile Paranoid Dementia OR Dementias, Senile Paranoid OR Paranoid Dementia, Senile OR Paranoid Dementias, Senile OR Senile Paranoid Dementias OR	18080

	Familial Dementia OR Dementia, Familial OR Dementias, Familial OR Familial Dementias OR demention OR dementia):ti,ab,kw	
4	1 AND 2 AND 3	15

Web of science		
No.	Query	Results
1	TS=(beta-Glucocerebrosidase OR beta Glucocerebrosidase OR Acid beta-Glucosidase OR Acid beta Glucosidase OR beta-Glucosidase, Acid OR Glucocerebroside beta-Glucosidase OR Glucocerebroside beta Glucosidase OR beta-Glucosidase, Glucocerebroside OR Glucosylceramide beta-Glucosidase OR Glucosylceramide beta Glucosidase OR beta-Glucosidase, Glucosylceramide OR Glucosylsphingosine Glucosyl Hydrolase OR Glucosyl Hydrolase, Glucosylsphingosine OR Hydrolase, Glucosylsphingosine Glucosyl OR Glucosyl Ceramidase OR Ceramidase, Glucosyl OR Glucocerebrosidase OR beta glucosylceramide glucosidase OR d glucosyl n acylsphingosine glucohydrolase OR e.c. 3.2.1.45 OR glucosylceramide glucosidase OR recombinant human derived r-glucacerebrosidase (r-gcr) OR glucosylceramidase)	23183
2	TS=(Idiopathic Parkinson's Disease OR Lewy Body Parkinson's Disease OR Parkinson's Disease, Idiopathic OR Parkinson's Disease, Lewy Body OR Parkinson Disease, Idiopathic OR Parkinson's Disease OR Idiopathic Parkinson Disease OR Lewy Body Parkinson Disease OR Primary Parkinsonism OR Parkinsonism, Primary OR Paralysis Agitans OR idiopathic parkinsonism OR Lewy bodies of Parkinson disease OR Lewy bodies of Parkinson`s disease OR Lewy bodies of Parkinsons disease OR Lewy body Parkinson disease OR Lewy body Parkinson`s disease OR Lewy body Parkinsons disease OR paralysis agitans OR Parkinson dementia complex OR Parkinsons disease OR primary parkinsonism OR Parkinson disease)	345470
3	TS=(Dementias OR Amentia OR Amentias OR Senile Paranoid Dementia OR Dementias, Senile Paranoid OR Paranoid Dementia, Senile OR Paranoid Dementias, Senile OR Senile Paranoid Dementias OR Familial Dementia OR Dementia, Familial OR Dementias, Familial OR Familial Dementias OR demention OR dementia)	381074
4	1 AND 2 AND 3	626

Embase		
No.	Query	Results
1	‘beta-Glucocerebrosidase’:ab,ti OR ‘beta Glucocerebrosidase’:ab,ti OR ‘Acid beta-Glucosidase’:ab,ti OR ‘Acid beta Glucosidase’:ab,ti OR ‘beta-Glucosidase, Acid’:ab,ti OR ‘Glucocerebroside beta-Glucosidase’:ab,ti OR ‘Glucocerebroside beta Glucosidase’:ab,ti OR ‘beta-Glucosidase, Glucocerebroside’:ab,ti OR ‘Glucosylceramide beta-Glucosidase’:ab,ti OR ‘Glucosylceramide beta Glucosidase’:ab,ti OR ‘beta-Glucosidase, Glucosylceramide’:ab,ti OR ‘Glucosylsphingosine Glucosyl Hydrolase’:ab,ti OR ‘Glucosyl Hydrolase, Glucosylsphingosine’:ab,ti OR ‘Hydrolase, Glucosylsphingosine Glucosyl’:ab,ti OR ‘Glucosyl Ceramidase’:ab,ti OR ‘Ceramidase, Glucosyl’:ab,ti OR ‘Glucocerebrosidase ’ :ab,ti OR ‘ beta glucosylceramide glucosidase’:ab,ti OR ‘d glucosyl n acylsphingosine glucohydrolase’:ab,ti OR ‘e.c. 3.2.1.45’:ab,ti OR ‘glucosylceramide glucosidase’:ab,ti OR ‘recombinant human derived r-glucacerebrosidase (r-gcr)’:ab,ti OR ‘glucosylceramidase’:ab,ti	4078
2	'idiopathic parkinsons disease':ab,ti OR 'parkinsons disease, idiopathic':ab,ti OR 'parkinsons disease, lewy body':ab,ti OR 'parkinson disease, idiopathic':ab,ti OR 'idiopathic parkinson disease':ab,ti OR 'parkinsonism, primary':ab,ti OR 'idiopathic parkinsonism':ab,ti OR 'lewy bodies of parkinson disease':ab,ti OR 'lewy bodies of parkinson`s disease':ab,ti OR 'lewy bodies of parkinsons disease':ab,ti OR 'lewy body parkinson disease':ab,ti OR 'lewy body parkinson`s disease':ab,ti OR 'lewy body parkinsons disease':ab,ti OR 'paralysis agitans':ab,ti OR 'parkinson dementia complex':ab,ti OR 'parkinsons disease':ab,ti OR 'primary parkinsonism':ab,ti OR 'parkinson disease':ab,ti	21316
3	dementias:ab,ti OR amentia:ab,ti OR amentias:ab,ti OR 'senile paranoid dementia':ab,ti OR 'dementias, senile paranoid':ab,ti OR 'paranoid dementia, senile':ab,ti OR 'paranoid dementias, senile':ab,ti OR 'senile paranoid dementias':ab,ti OR 'familial dementia':ab,ti OR 'dementia, familial':ab,ti OR 'dementias, familial':ab,ti OR 'familial dementias':ab,ti OR demention:ab,ti OR dementia:ab,ti	211957
4	1 AND 2 AND 3	56
