# Supplementary material for: Effects of glucocerebrosidase gene variations on the risk of Parkinson’s disease dementia: a meta-analysis
Source: Front Aging Neurosci. 2025 Nov 14;17:1671760. doi: 10.3389/fnagi.2025.1671760 (PMC12660284; doi:10.3389/fnagi.2025.1671760)

Supplementary Table 1. Characteristics of included studies and patients

Author, year	Country	PD diagnostic criteria	Dementia evaluation method	Study design	group	sample size	Male%	Age at onset (Mean±SD)	Disease duration (Mean ± SD)	*key findings of each included study*
**Agosta, 2013(11)**	**Italy**	**UK Brain Bank criteria**	**MDS**	**Cohort study**	**A**	**15**	**60.0**	**54±7**	**10±6**	Parkinson’s disease patients carrying glucocerebrosidase gene mutations experience a distributed pattern of white matter abnormalities involving the interhemispheric, frontal corticocortical, and parahippocampal tracts. White matter pathology in these patients may have an impact on the clinical manifestations of the disease, including cognitive impairment.
					**B**	**14**	**57.1**	**53±8**	**11±6**	
**Alcalay, 2012(19)**	**USA**	**UK Brain Bank criteria**	**CDR、MMSE**	**Cohort study**	**A**	**26**	**69.2**	**42.9±5.2**	**15.4±5.8**	GBA mutation status may be an independent risk factor for cognitive impairment in patients with PD.
					**B**	**39**	**59.0**	**43.6±4.9**	**14.7±5.4**	
**Chen, 2023(31)**	**China**	**UK Brain Bank criteria**	**MDS**	**Cohort study**	**A**	**36**	**NA**	**NA**	**NA**	In NDPD, the development of CI during the 5-year follow-up can be predicted with good accuracy using a model combining age, current diagnosis of hypertension, baseline MoCA scores, MDS-UPDRS III scores, and APOE status. Our study underscores the need for the earlier identification of CI in NDPD patients in our clinical practice.
					**B**	**196**	**NA**	**NA**	**NA**	

**Cilia, 2016(32)**	**Italy**	**UK Brain Bank criteria**	**DSM-IV、MMSE**	**Cohort study**	**A**	**123**	**56.1**	**52.4±10.2**	**11.9±6.3**	Survival is reduced in GBA-carriers compared to non-carriers; this seems to be partially independent from the increased risk for early dementia. The risk for dementia is strongly modulated by the type of mutation. In the clinical continuum between PD and DLB, patients with GBA mutations seem to localize midway, with carriers of severe mutations closer to DLB than to idiopathic PD.
					**B**	**1982**	**60.8**	**57.4±10.6**	**12.0±6.6**	
**Davis, 2016(33)**	**USA**	**UK Brain Bank criteria**	**MDS**	**Cohort study**	**A**	**58**	**55.2**	**NA**	**8.4±5.2**	GBA variants predict a more rapid progression of cognitive dysfunction and motor symptoms in patients with PD, with a greater effect on PIGD than tremor. Thus, GBA variants influence the heterogeneity in symptom progression observe in PD.
					**B**	**675**	**70.4**	**NA**	**8.7±6.1**	
**De Michele, 2023(34)**	**Italy**	**UK Brain Bank criteria**	**MDS**	**Cohort study**	**A**	**11**	**36.4**	**NA**	**9.2±4.8**	Our results confirm that NMS and a more severe and faster disease course more frequently occur among GBAPD patients in comparison to iPD.
					**B**	**22**	**36.4**	**NA**	**8.8±4.5**	
**Graham, 2020(35)**	**New Zealand**	**UK Brain Bank criteria**	**MDS**	**Cohort study**	**A**	**21**	**71**	**58.3±9.2**	**15.2±6.9**	This work confirmed the utility of nanopore sequencing as a high-throughput method to identify known and novel GBA variants, and to assign precise haplotypes. Our observations may contribute to improved understanding of the effects of variants on disease pathogenesis, and to the development of more targeted treatments.
					**B**	**208**	**67.3**	**60.7±8.5**	**13.7±5.8**	
**Lunde, 2018(36)**	**Norway**	**UK Brain Bank criteria**	**MDS**	**Cohort study**	**A**	**53**	**64.2**	**64.98±9.79**	**NA**	GBA variants are of great clinical relevance for the development of dementia in Parkinson’s disease, especially due to the relatively higher frequency of these alleles compared with other risk alleles.
					**B**	**389**	**59.9**	**68.03±9.63**	**NA**	
**Malec-Litwinowicz, 2014(2)**	**Poland**	**UK Brain Bank criteria**	**MMSE**	**Cohort study**	**A**	**5**	**NA**	**57.2±2.8**	**NA**	The N370S GBA mutation is the risk factor for cognitive impairment in PD patients.
					**B**	**117**	**NA**	**57.6±10.9**	**NA**	

**Malek, 2018(37)**	**UK**	**UK Brain Bank criteria**	**MDS**	**Cohort study**	**A**	**142**	**65.5**	**64.3±10.1**	**1.3±1.0**	Our study confirms the influence of GBA mutations on the age of onset, disease severity and motor phenotype in patients with PD. Cognition did not differ between GBA mutation carriers and noncarriers at baseline, implying that cognitive impairment/ dementia, reported in other studies at a later disease stage, is not present in recently diagnosed cases. This offers an important window of opportunity for potential disease-modifying therapy that may protect against the development of dementia in GBA-PD.
					**B**	**1584**	**65.4**	**66.2±9.2**	**1.3±0.9**	
**Mata, 2016(38)**	**USA**	**UK Brain Bank criteria**	**MDS**	**Cohort study**	**A**	**95**	**NA**	**55.8±10.7**	**NA**	Both GBA mutations and E326K are associated with a distinct cognitive profile characterized by greater impairment in working memory/executive function and visuospatial abilities in PD patients. The discovery that E326K negatively impacts cognitive performance approximately doubles the proportion of PD patients we now recognize are at risk for more severe GBA-related cognitive deficits.
					**B**	**945**	**NA**	**59.7±10.5**	**NA**	
**Moran, 2017(39)**	**USA**	**UK Brain Bank criteria**	**DSM-IV**	**Cohort study**	**A**	**28**	**39**	**NA**	**NA**	Taken together, these results suggest an effect, but an overall limited burden, of harboring a single GBA mutation in aging mutation carriers
					**B**	**708**	**35.7**	**NA**	**NA**	
**Oeda, 2015(40)**	**Japan**	**UK Brain Bank criteria**	**DSM-IV**	**Cohort study**	**A**	**19**	**26.3**	**55.2±9.9**	**6.9±4.6**	N-isopropyl-p[123I] iodoamphetamine single-photon emission tomography pixel-by-pixel analysis revealed that regional cerebral blood flow was reduced in the bilateral parietal cortex, including the precuneus of GD-associated mutan PD patients, compared with matched PD controls without mutations.
					**B**	**196**	**50.0**	**59.4±11.5**	**7.6±5.4**	
**Seto´-Salvia, 2012(41)**	**Spain**	**UK Brain Bank criteria**	**CDR、DSM-IV**	**Cohort study**	**A**	**22**	**27.3**	**54.2±6.6**	**14.1±6.5**	Our findings suggest that mutations in the glucocerebrosidase gene not only increase the risk of both Parkinson’s disease and Lewy body dementia but also strongly influence the course of Parkinson’s disease with respect to the appearance of dementia.
					**B**	**203**	**56.7**	**56.5±12.7**	**12.0±6.7**	

**Simuni, 2020(42)**	**USA**	**UK Brain Bank criteria**	**MDS**	**Cohort study**	**A**	**80**	**53.8**	**58.4±10.7**	**3.1±2.0**	We confirm previous reports of milder phenotype associated with LRRK2-PD. A previously reported more aggressive phenotype in GBA-PD is not evident early in the disease in N370s carriers. This observation identifies a window for potential disease-modifying interventions. Longitudinal data will be essential to define the slope of progression for both genetic cohorts.
					**B**	**361**	**65.9**	**59.7±9.9**	**2.6±0.6**	
**Straniero, 2020(43)**	**Italy**	**UK Brain Bank criteria**	**DSM-IV、MDS**	**Cohort study**	**A**	**248**	**54.44**	**54.35±10.96**	**14.05±7.29**	We report a large monocentric study on GBA-PD assessing mutation-specific dat on the sex distribution, penetrance, incidence, and association with dementia of the 4 most frequent deleterious variants in GBA.
					**B**	**3433**	**60.30**	**58.24±10.78**	**13.55±6.89**	
**Szwedo, 2022(44)**	**Norway**	**UK Brain Bank criteria**	**DSM-IV、MDS**	**Cohort study**	**A**	**100**	**63.7**	**65.3±9.4**	**NA**	GBA and APOE genotyping could improve the prediction of cognitive decline in PD, which is important to inform the clinical trial selection and potentially to enable personalized treatment
					**B**	**867**	**60.8**	**67.6±9.9**	**NA**	
**Yahalom, 2019(45)**	**Israel**	**UK Brain Bank criteria**	**MoCA、MMSE**	**Cohort study**	**A**	**76**	**60.3**	**58.6±10.0**	**11.5±7.0**	While GBA-PD is characterized by higher rates of dementia, probable RBD and psychosis, it seems that compared to the other groups, these features are less common for LRRK2-GBA-PD. This may imply to a possible protective effect of LRRK2 p.G2019S mutation among GBA variant carriers.
					**B**	**78**	**65.0**	**61.4±11.7**	**11.3±6.5**	

A: Parkinson's disease patients with GBA variation; B: Parkinson's disease patients without GBA variation; NA: not available; MDS: Movement Disorder Society criteria; CDR: Clinical Dementia Rating; MMSE: Mini–Mental State Examination; DSM-IV: Diagnostic and Statistical Manual of Mental Disorders, Fourth Edition; MoCA: Montreal Cognitive Assessment.

Supplement Figure 1. Forest plot of subgroup analysis for GBA mutations by ethnicity

Study or Subgroup	Experimental Events	Experimental Total	Control Events	Control Total	Weight	Risk Ratio IV, Random, 95% CI
**1.2.1 Asian**						
Chen 2023	17	36	77	196	9.2%	1.20 [0.82, 1.77]
Oeda 2015	9	19	34	196	6.8%	2.73 [1.55, 4.80]
**Subtotal (95% CI)**		**55**		**392**	**16.0%**	**1.76 [0.79, 3.94]**
Total events	26		111			
Heterogeneity: Tau² = 0.28; Chi² = 5.53, df = 1 (P = 0.02); I² = 82%						
Test for overall effect: Z = 1.39 (P = 0.17)						
**1.2.2 Caucasian**						
Agosta 2013	9	15	0	14	0.6%	17.81 [1.13, 280.09]
Alcalay 2012	11	26	6	39	4.2%	2.75 [1.16, 6.51]
Cilia 2016	42	123	388	1982	11.0%	1.74 [1.34, 2.26]
Davis 2016	9	27	58	675	6.6%	3.88 [2.16, 6.98]
De Michele 2023	2	3	0	22	0.5%	28.75 [1.67, 494.38]
Lunde 2018	4	8	107	389	5.3%	1.82 [0.89, 3.70]
Malec-Litwinowicz 2014	3	5	23	117	4.6%	3.05 [1.37, 6.82]
Malek 2018	8	44	376	1584	6.1%	0.77 [0.41, 1.44]
Mata 2015	16	48	143	945	8.6%	2.20 [1.44, 3.38]
Seto´-Salvia 2011	11	22	48	203	7.8%	2.11 [1.30, 3.44]
Simuni 2020	10	80	58	361	6.2%	0.78 [0.42, 1.45]
Straniero 2020	53	138	717	3433	11.5%	1.84 [1.47, 2.29]
Szwedo 2022	42	100	239	867	11.1%	1.52 [1.18, 1.96]
**Subtotal (95% CI)**		**639**		**10631**	**84.0%**	**1.86 [1.48, 2.34]**
Total events	220		2163			
Heterogeneity: Tau² = 0.09; Chi² = 32.34, df = 12 (P = 0.001); I² = 63%						
Test for overall effect: Z = 5.27 (P < 0.00001)						
**Total (95% CI)**		**694**		**11023**	**100.0%**	**1.83 [1.48, 2.27]**
Total events	246		2274			
Heterogeneity: Tau² = 0.09; Chi² = 38.44, df = 14 (P = 0.0004); I² = 64%						
Test for overall effect: Z = 5.59 (P < 0.00001)						
Test for subgroup differences: Chi² = 0.02, df = 1 (P = 0.90), I² = 0%						

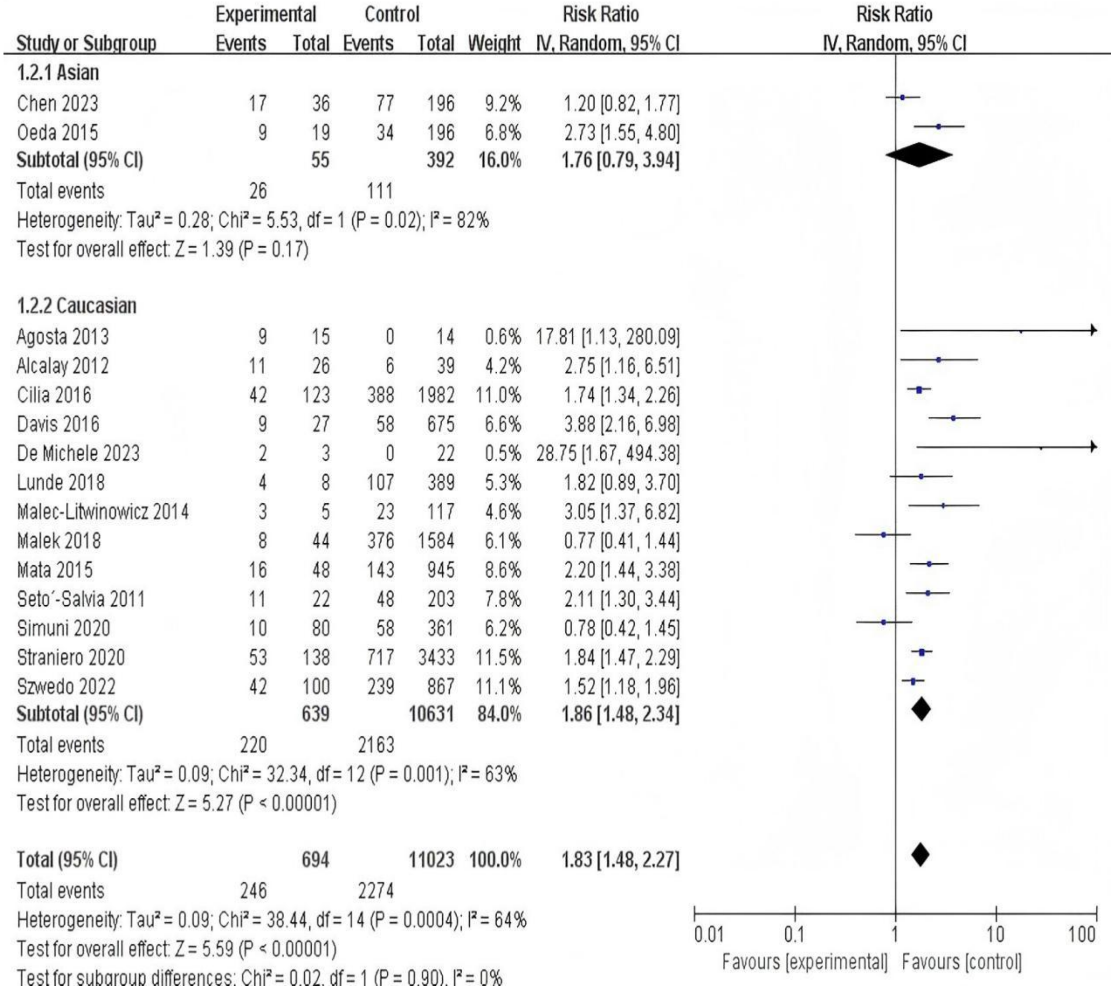

Supplement Figure 2. Forest plot of subgroup analysis for GBA variations by ethnicity

Study or Subgroup	Experimental Events	Experimental Total	Control Events	Control Total	Weight	Risk Ratio IV, Random, 95% CI
**2.2.1 Asian**						
Chen 2023	17	36	77	196	7.4%	1.20 [0.82, 1.77]
Oeda 2015	9	19	34	196	5.5%	2.73 [1.55, 4.80]
**Subtotal (95% CI)**		**55**		**392**	**12.9%**	**1.76 [0.79, 3.94]**
Total events	26		111			
Heterogeneity: Tau² = 0.28; Chi² = 5.53, df = 1 (P = 0.02); I² = 82%						
Test for overall effect: Z = 1.39 (P = 0.17)						
**2.2.2 Caucasian**						
Agosta 2013	9	15	0	14	0.4%	17.81 [1.13, 280.09]
Alcalay 2012	11	26	6	39	3.3%	2.75 [1.16, 6.51]
Cilia 2016	42	123	388	1982	8.9%	1.74 [1.34, 2.26]
Davis 2016	16	58	58	675	6.3%	3.21 [1.98, 5.21]
De Michele 2023	5	11	0	22	0.4%	21.08 [1.27, 350.08]
Lunde 2018	22	53	107	389	7.7%	1.51 [1.05, 2.16]
Malec-Litwinowicz 2014	3	5	23	117	3.6%	3.05 [1.37, 6.82]
Malek 2018	32	142	376	1584	8.2%	0.95 [0.69, 1.30]
Mata 2015	35	95	143	945	8.4%	2.43 [1.80, 3.30]
Seto´-Salvia 2011	11	22	48	203	6.3%	2.11 [1.30, 3.44]
Simuni 2020	10	80	58	361	4.9%	0.78 [0.42, 1.45]
Straniero 2020	90	248	717	3433	9.8%	1.74 [1.46, 2.07]
Szwedo 2022	42	100	239	867	9.0%	1.52 [1.18, 1.96]
Yahalom 2018	24	76	9	78	4.3%	2.74 [1.36, 5.50]
**Subtotal (95% CI)**		**1054**		**10709**	**81.7%**	**1.84 [1.49, 2.27]**
Total events	352		2172			
Heterogeneity: Tau² = 0.09; Chi² = 43.77, df = 13 (P < 0.0001); I² = 70%						
Test for overall effect: Z = 5.65 (P < 0.00001)						
**2.2.3 Oceanian**						
Graham 2019	9	21	42	208	5.5%	2.12 [1.21, 3.73]
**Subtotal (95% CI)**		**21**		**208**	**5.5%**	**2.12 [1.21, 3.73]**
Total events	9		42			
Heterogeneity: Not applicable						
Test for overall effect: Z = 2.62 (P = 0.009)						
**Total (95% CI)**		**1130**		**11309**	**100.0%**	**1.83 [1.52, 2.21]**
Total events	387		2325			
Heterogeneity: Tau² = 0.09; Chi² = 50.19, df = 16 (P < 0.0001); I² = 68%						
Test for overall effect: Z = 6.31 (P < 0.00001)						
Test for subgroup differences: Chi² = 0.24, df = 2 (P = 0.89), I² = 0%						

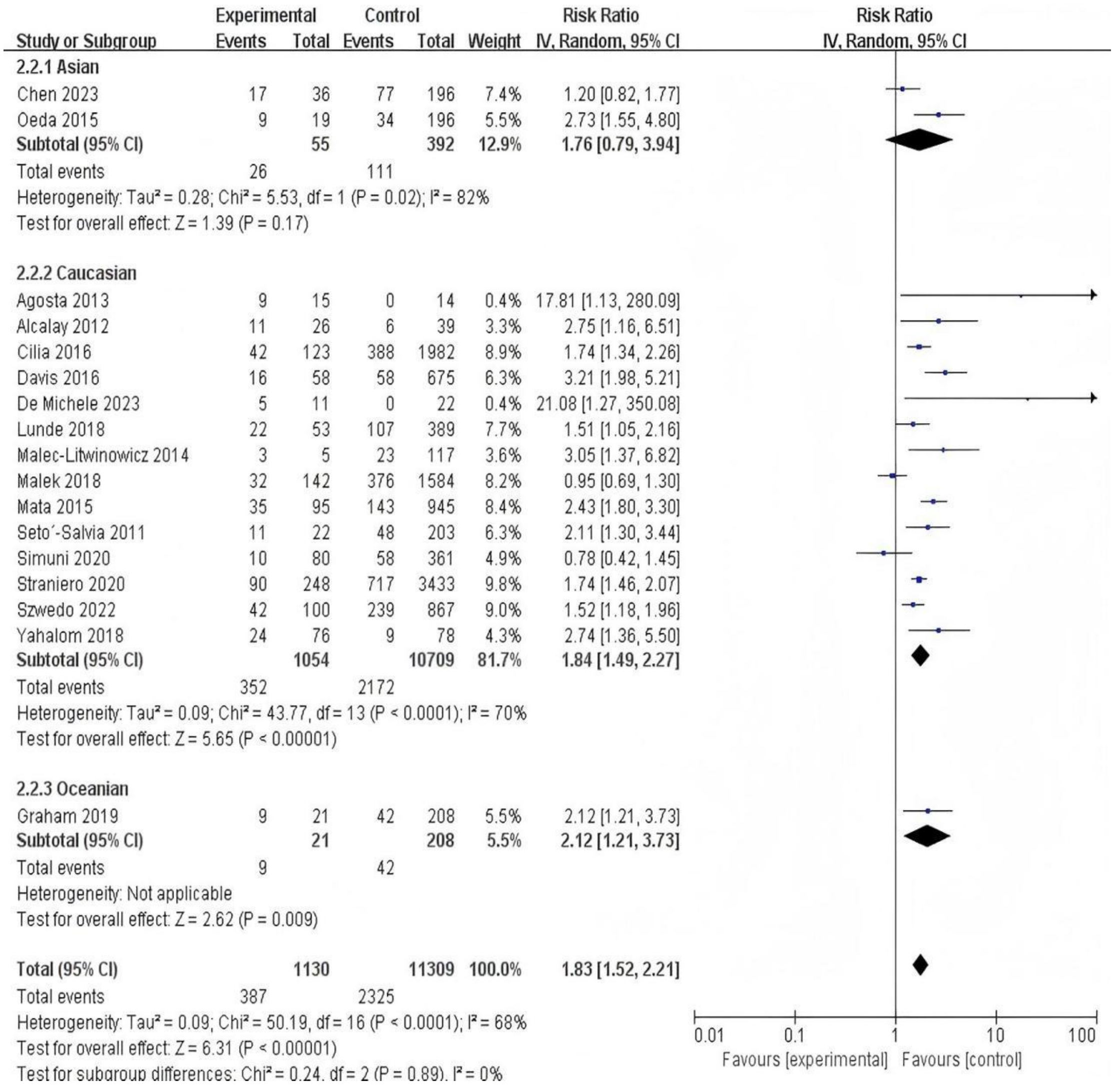

Supplement Figure 3. Forest plot of subgroup analysis for GBA mutations by dementia diagnostic criteria

Study or Subgroup	Experimental Events	Experimental Total	Control Events	Control Total	Weight	Risk Ratio IV, Random, 95% CI
**1.3.1 CDR**						
Alcalay 2012	11	26	6	39	2.1%	2.75 [1.16, 6.51]
Seto´-Salvia 2011	11	22	48	203	4.8%	2.11 [1.30, 3.44]
**Subtotal (95% CI)**		**48**		**242**	**6.9%**	**2.25 [1.48, 3.44]**
Total events	22		54			
Heterogeneity: Tau² = 0.00; Chi² = 0.27, df = 1 (P = 0.60); I² = 0%						
Test for overall effect: Z = 3.76 (P = 0.0002)						
**1.3.2 DSM-IV**						
Cilia 2016	42	123	388	1982	8.2%	1.74 [1.34, 2.26]
Moran 2017	7	28	107	708	3.2%	1.65 [0.85, 3.22]
Oeda 2015	9	19	34	196	4.0%	2.73 [1.55, 4.80]
Seto´-Salvia 2011	11	22	48	203	4.8%	2.11 [1.30, 3.44]
Straniero 2020	53	138	717	3433	8.9%	1.84 [1.47, 2.29]
Szwedo 2022	42	100	239	867	8.3%	1.52 [1.18, 1.96]
**Subtotal (95% CI)**		**430**		**7389**	**37.5%**	**1.78 [1.56, 2.02]**
Total events	164		1533			
Heterogeneity: Tau² = 0.00; Chi² = 4.28, df = 5 (P = 0.51); I² = 0%						
Test for overall effect: Z = 8.74 (P < 0.00001)						
**1.3.3 MDS**						
Agosta 2013	9	15	0	14	0.2%	17.81 [1.13, 280.09]
Chen 2023	17	36	77	196	6.1%	1.20 [0.82, 1.77]
Davis 2016	9	27	58	675	3.8%	3.88 [2.16, 6.98]
De Michele 2023	2	3	0	22	0.2%	28.75 [1.67, 494.38]
Lunde 2018	4	8	107	389	2.9%	1.82 [0.89, 3.70]
Malek 2018	8	44	376	1584	3.4%	0.77 [0.41, 1.44]
Mata 2015	16	48	143	945	5.5%	2.20 [1.44, 3.38]
Simuni 2020	10	80	58	361	3.5%	0.78 [0.42, 1.45]
Straniero 2020	53	138	717	3433	8.9%	1.84 [1.47, 2.29]
Szwedo 2022	42	100	239	867	8.3%	1.52 [1.18, 1.96]
**Subtotal (95% CI)**		**499**		**8486**	**42.9%**	**1.65 [1.23, 2.23]**
Total events	170		1775			
Heterogeneity: Tau² = 0.13; Chi² = 31.77, df = 9 (P = 0.0002); I² = 72%						
Test for overall effect: Z = 3.30 (P = 0.0010)						
**1.3.4 MMSE**						
Alcalay 2012	11	26	6	39	2.1%	2.75 [1.16, 6.51]
Cilia 2016	42	123	388	1982	8.2%	1.74 [1.34, 2.26]
Malec-Litwinowicz 2014	3	5	23	117	2.4%	3.05 [1.37, 6.82]
**Subtotal (95% CI)**		**154**		**2138**	**12.7%**	**2.02 [1.44, 2.82]**
Total events	56		417			
Heterogeneity: Tau² = 0.02; Chi² = 2.45, df = 2 (P = 0.29); I² = 18%						
Test for overall effect: Z = 4.11 (P < 0.0001)						
**Total (95% CI)**		**1131**		**18255**	**100.0%**	**1.80 [1.57, 2.07]**
Total events	412		3779			
Heterogeneity: Tau² = 0.04; Chi² = 41.44, df = 20 (P = 0.003); I² = 52%						
Test for overall effect: Z = 8.29 (P < 0.00001)						
Test for subgroup differences: Chi² = 1.87, df = 3 (P = 0.60), I² = 0%						

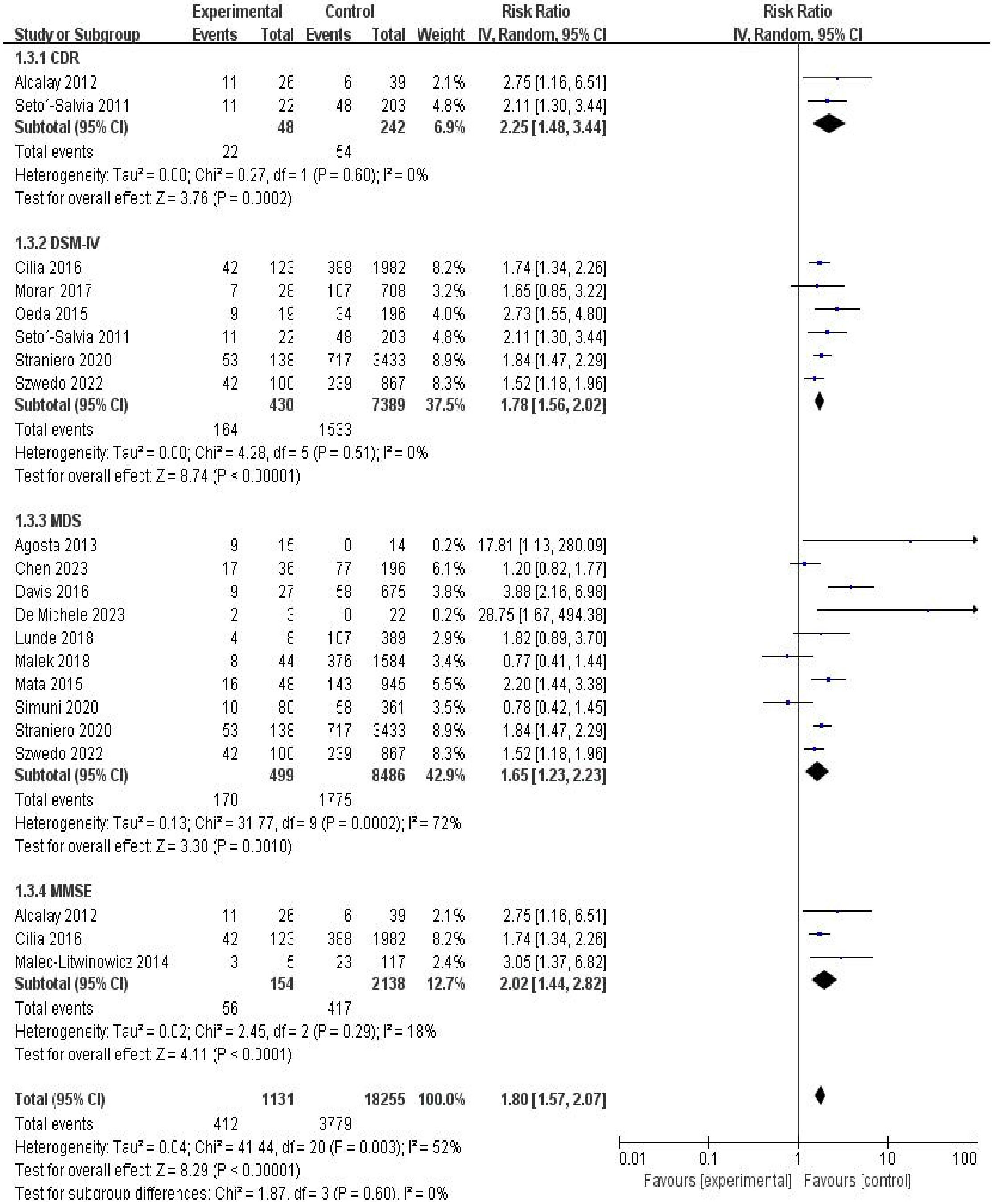

Supplement Figure 4. Forest plot of subgroup analysis for GBA variations by dementia diagnostic criteria

Study or Subgroup	Experimental Events	Experimental Total	Control Events	Control Total	Weight	Risk Ratio IV, Random, 95% CI
**2.3.1 CDR**						
Alcalay 2012	11	26	6	39	1.8%	2.75 [1.16, 6.51]
Seto´-Salvia 2011	11	22	48	203	4.0%	2.11 [1.30, 3.44]
**Subtotal (95% CI)**		**48**		**242**	**5.8%**	**2.25 [1.48, 3.44]**
Total events	22		54			
Heterogeneity: Tau² = 0.00; Chi² = 0.27, df = 1 (P = 0.60); I² = 0%						
Test for overall effect: Z = 3.76 (P = 0.0002)						
**2.3.2 DSM-IV**						
Cilia 2016	42	123	388	1982	6.7%	1.74 [1.34, 2.26]
Moran 2017	7	28	107	708	2.7%	1.65 [0.85, 3.22]
Oeda 2015	9	19	34	196	3.3%	2.73 [1.55, 4.80]
Seto´-Salvia 2011	11	22	48	203	4.0%	2.11 [1.30, 3.44]
Straniero 2020	90	248	717	3433	7.8%	1.74 [1.46, 2.07]
Szwedo 2022	42	100	239	867	6.8%	1.52 [1.18, 1.96]
**Subtotal (95% CI)**		**540**		**7389**	**31.2%**	**1.74 [1.55, 1.96]**
Total events	201		1533			
Heterogeneity: Tau² = 0.00; Chi² = 4.14, df = 5 (P = 0.53); I² = 0%						
Test for overall effect: Z = 9.20 (P < 0.00001)						
**2.3.3 MDS**						
Agosta 2013	9	15	0	14	0.2%	17.81 [1.13, 280.09]
Chen 2023	17	36	77	196	5.0%	1.20 [0.82, 1.77]
Davis 2016	16	58	58	675	4.0%	3.21 [1.98, 5.21]
De Michele 2023	5	11	0	22	0.2%	21.08 [1.27, 350.08]
Graham 2019	9	21	42	208	3.3%	2.12 [1.21, 3.73]
Lunde 2018	22	53	107	389	5.4%	1.51 [1.05, 2.16]
Malek 2018	32	142	376	1584	5.9%	0.95 [0.69, 1.30]
Mata 2015	35	95	143	945	6.1%	2.43 [1.80, 3.30]
Simuni 2020	10	80	58	361	2.9%	0.78 [0.42, 1.45]
Straniero 2020	90	248	717	3433	7.8%	1.74 [1.46, 2.07]
Szwedo 2022	42	100	239	867	6.8%	1.52 [1.18, 1.96]
**Subtotal (95% CI)**		**859**		**8694**	**47.6%**	**1.65 [1.29, 2.11]**
Total events	287		1817			
Heterogeneity: Tau² = 0.11; Chi² = 41.00, df = 10 (P < 0.0001); I² = 76%						
Test for overall effect: Z = 3.95 (P < 0.0001)						
**2.3.4 MMSE**						
Alcalay 2012	11	26	6	39	1.8%	2.75 [1.16, 6.51]
Cilia 2016	42	123	388	1982	6.7%	1.74 [1.34, 2.26]
Malec-Litwinowicz 2014	3	5	23	117	2.0%	3.05 [1.37, 6.82]
Yahalom 2018	24	76	9	78	2.5%	2.74 [1.36, 5.50]
**Subtotal (95% CI)**		**230**		**2216**	**13.0%**	**2.07 [1.57, 2.72]**
Total events	80		426			
Heterogeneity: Tau² = 0.01; Chi² = 3.40, df = 3 (P = 0.33); I² = 12%						
Test for overall effect: Z = 5.15 (P < 0.00001)						
**2.3.5 MoCA**						
Yahalom 2018	24	76	9	78	2.5%	2.74 [1.36, 5.50]
**Subtotal (95% CI)**		**76**		**78**	**2.5%**	**2.74 [1.36, 5.50]**
Total events	24		9			
Heterogeneity: Not applicable						
Test for overall effect: Z = 2.83 (P = 0.005)						
**Total (95% CI)**		**1753**		**18619**	**100.0%**	**1.81 [1.59, 2.06]**
Total events	614		3839			
Heterogeneity: Tau² = 0.05; Chi² = 54.62, df = 23 (P = 0.0002); I² = 58%						
Test for overall effect: Z = 9.00 (P < 0.00001)						
Test for subgroup differences: Chi² = 4.23, df = 4 (P = 0.38), I² = 5.3%						

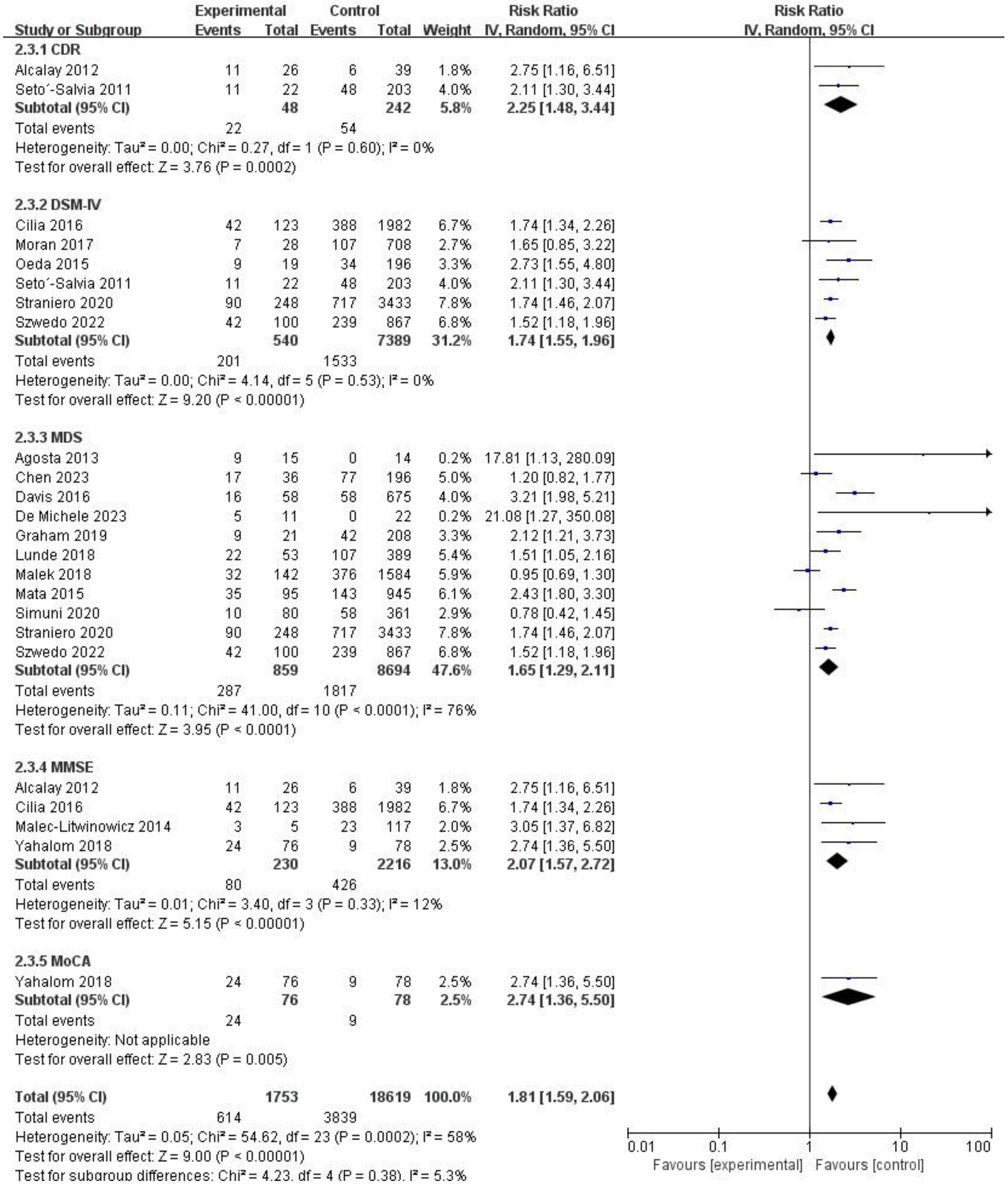

Supplement Figure 5. Forest plot of subgroup analysis for GBA polymorphisms by dementia diagnostic criteria

	Experimental		Control			Risk Ratio
Study or Subgroup	Events	Total	Events	Total	Weight	IV, Random, 95% CI
**1.2.1 DSM-IV**						
Straniero 2020	27	61	717	3433	19.4%	2.12 [1.59, 2.83]
**Subtotal (95% CI)**		**61**		**3433**	**19.4%**	**2.12 [1.59, 2.83]**
Total events	27		717			
Heterogeneity: Not applicable						
Test for overall effect: Z = 5.09 (P < 0.00001)						
**1.2.2 MDS**						
Davis 2016	7	31	58	675	10.0%	2.63 [1.31, 5.27]
Lunde 2018	18	45	107	389	16.6%	1.45 [0.98, 2.15]
Malek 2018	24	98	376	1584	17.5%	1.03 [0.72, 1.48]
Mata 2015	19	47	143	945	17.0%	2.67 [1.83, 3.90]
Straniero 2020	27	61	717	3433	19.4%	2.12 [1.59, 2.83]
**Subtotal (95% CI)**		**282**		**7026**	**80.6%**	**1.82 [1.26, 2.61]**
Total events	95		1401			
Heterogeneity: Tau² = 0.13; Chi² = 16.92, df = 4 (P = 0.002); I² = 76%						
Test for overall effect: Z = 3.22 (P = 0.001)						
**Total (95% CI)**		**343**		**10459**	**100.0%**	**1.86 [1.40, 2.48]**
Total events	122		2118			
Heterogeneity: Tau² = 0.09; Chi² = 17.91, df = 5 (P = 0.003); I² = 72%						
Test for overall effect: Z = 4.25 (P < 0.0001)						
Test for subgroup differences: Chi² = 0.43, df = 1 (P = 0.51), I² = 0%						

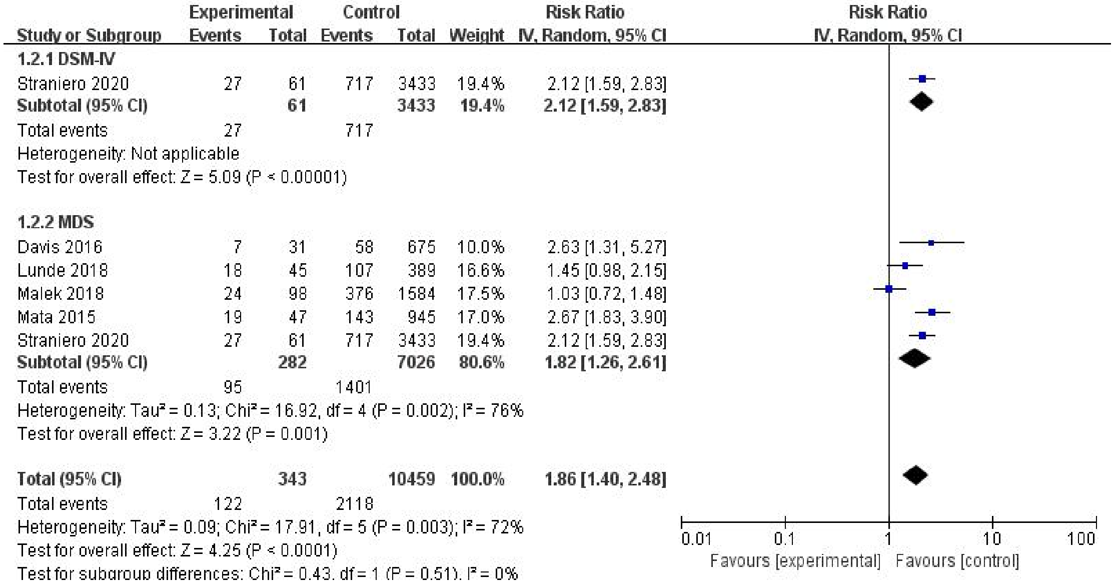

Supplement Figure 6. Forest plot of subgroup analysis for N370S mutation by dementia diagnostic criteria

	Experimental		Control			Risk Ratio
Study or Subgroup	Events	Total	Events	Total	Weight	IV, Random, 95% CI
**1.2.1 DSM-IV**						
Cilia 2016	17	67	388	1982	10.1%	1.30 [0.85, 1.97]
Straniero 2020	26	76	717	3433	17.5%	1.64 [1.19, 2.25]
Szwedo 2022	32	81	239	867	21.1%	1.43 [1.07, 1.92]
**Subtotal (95% CI)**		**224**		**6282**	**48.6%**	**1.47 [1.22, 1.78]**
Total events	75		1344			
Heterogeneity: Tau² = 0.00; Chi² = 0.82, df = 2 (P = 0.66); I² = 0%						
Test for overall effect: Z = 3.97 (P < 0.0001)						
**1.2.2 MDS**						
Straniero 2020	26	76	717	3433	17.5%	1.64 [1.19, 2.25]
Szwedo 2022	32	81	239	867	21.1%	1.43 [1.07, 1.92]
**Subtotal (95% CI)**		**157**		**4300**	**38.6%**	**1.52 [1.23, 1.89]**
Total events	58		956			
Heterogeneity: Tau² = 0.00; Chi² = 0.37, df = 1 (P = 0.54); I² = 0%						
Test for overall effect: Z = 3.84 (P = 0.0001)						
**1.2.3 MMSE**						
Cilia 2016	17	67	388	1982	10.1%	1.30 [0.85, 1.97]
Malec-Litwinowicz 2014	3	5	23	117	2.7%	3.05 [1.37, 6.82]
**Subtotal (95% CI)**		**72**		**2099**	**12.8%**	**1.85 [0.81, 4.24]**
Total events	20		411			
Heterogeneity: Tau² = 0.26; Chi² = 3.42, df = 1 (P = 0.06); I² = 71%						
Test for overall effect: Z = 1.46 (P = 0.14)						
**Total (95% CI)**		**453**		**12681**	**100.0%**	**1.50 [1.32, 1.72]**
Total events	153		2711			
Heterogeneity: Tau² = 0.00; Chi² = 4.70, df = 6 (P = 0.58); I² = 0%						
Test for overall effect: Z = 5.99 (P < 0.00001)						
Test for subgroup differences: Chi² = 0.30, df = 2 (P = 0.86), I² = 0%						

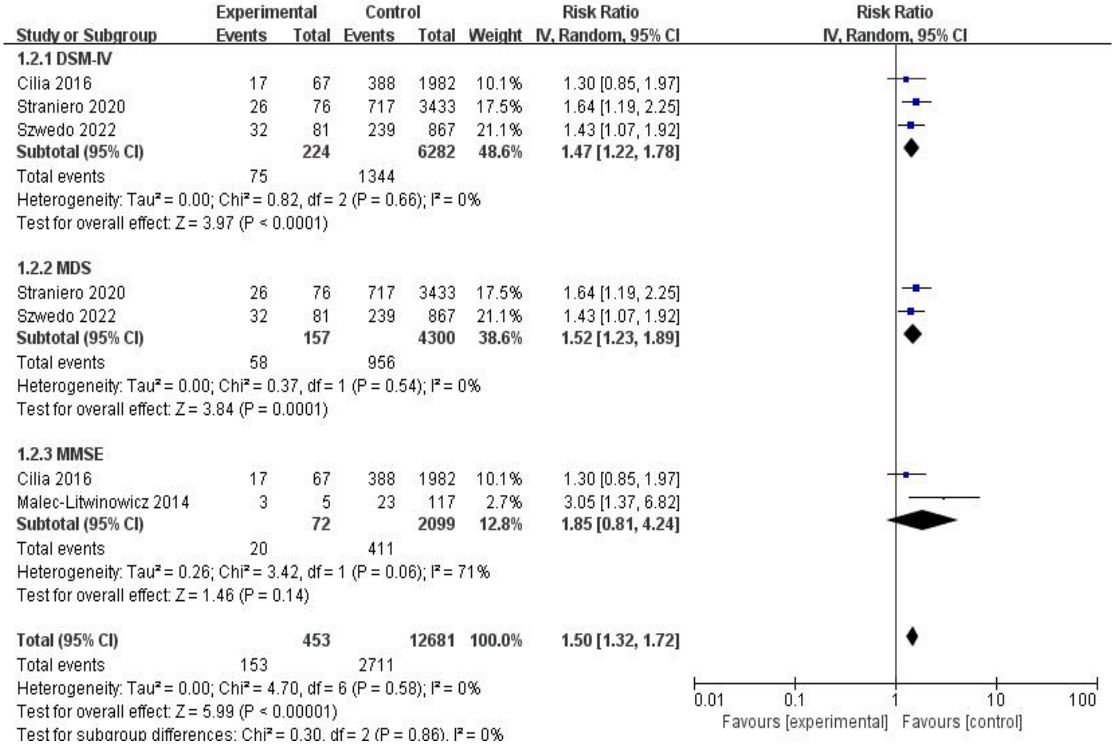

Supplement Figure 7. Forest plot of subgroup analysis for L444P mutation by dementia diagnostic criteria

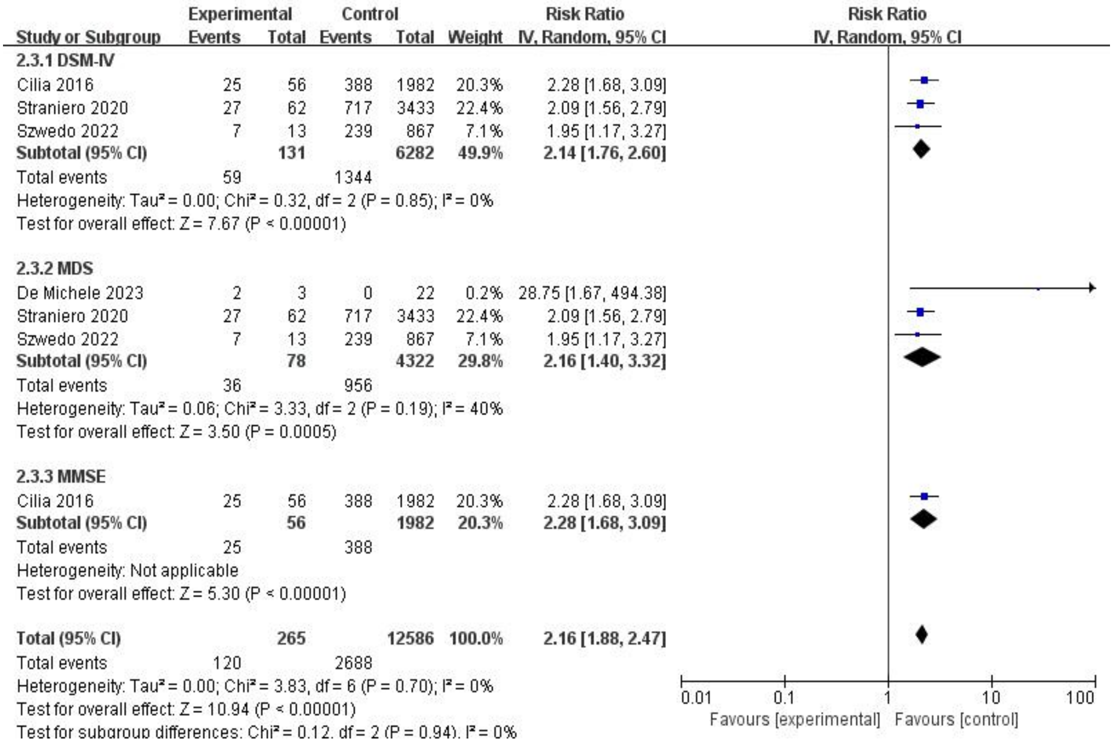

Supplement Figure 8. Forest plot of subgroup analysis for E326K polymorphism by dementia diagnostic criteria

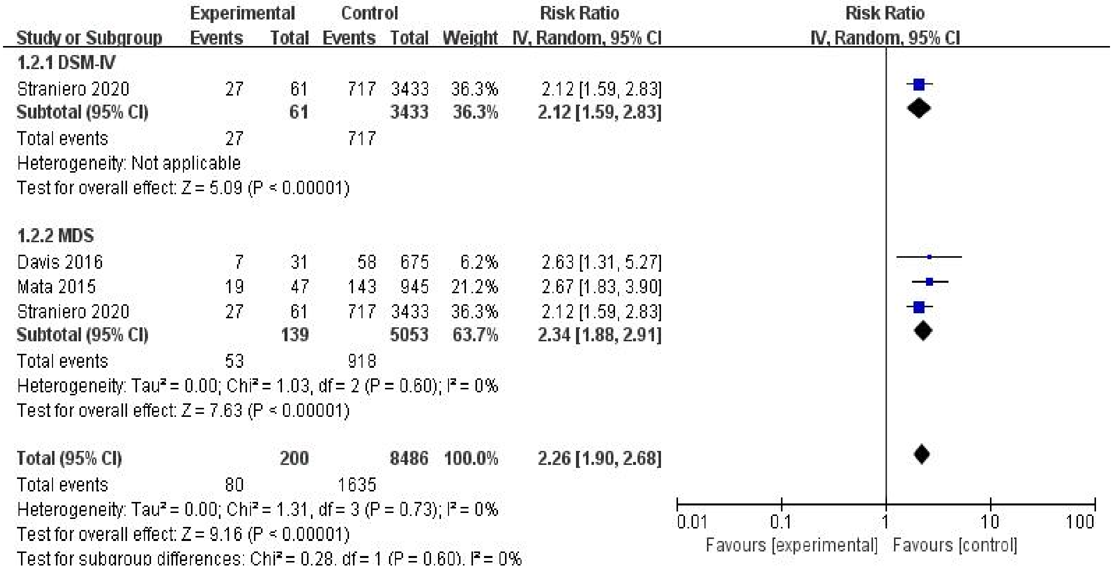

Supplement Figure 9. Sensitivity analysis for the overall association of GBA variations with dementia risk

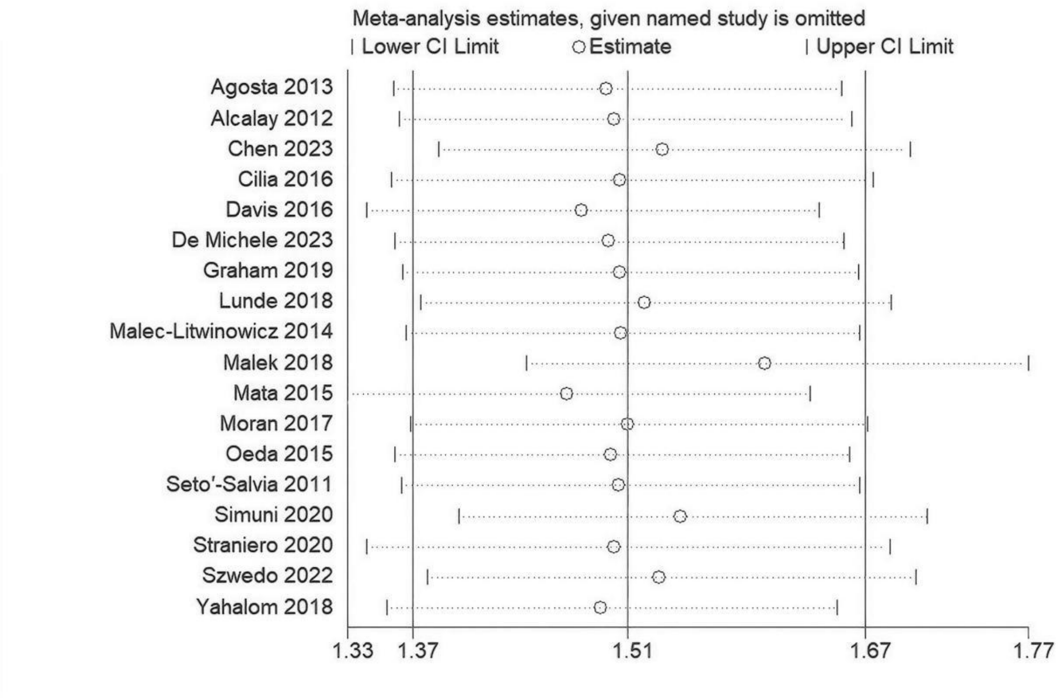

Supplement Figure 10. Sensitivity analysis for the association of GBA mutations with dementia risk

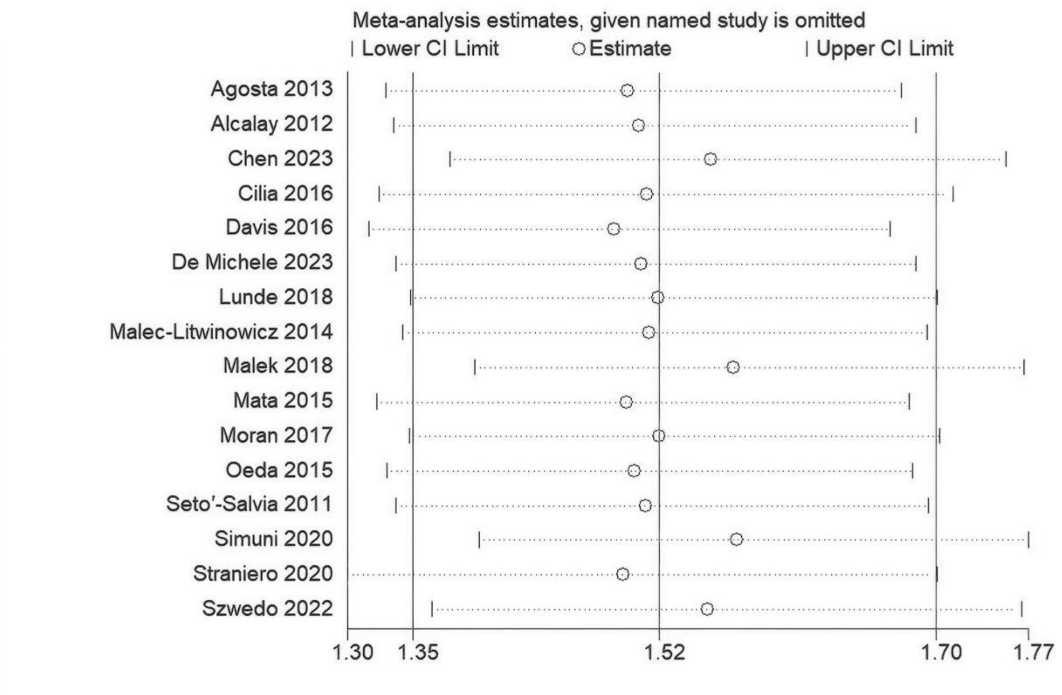

Supplement Figure 11. Sensitivity analysis for the association of GBA polymorphisms with dementia risk

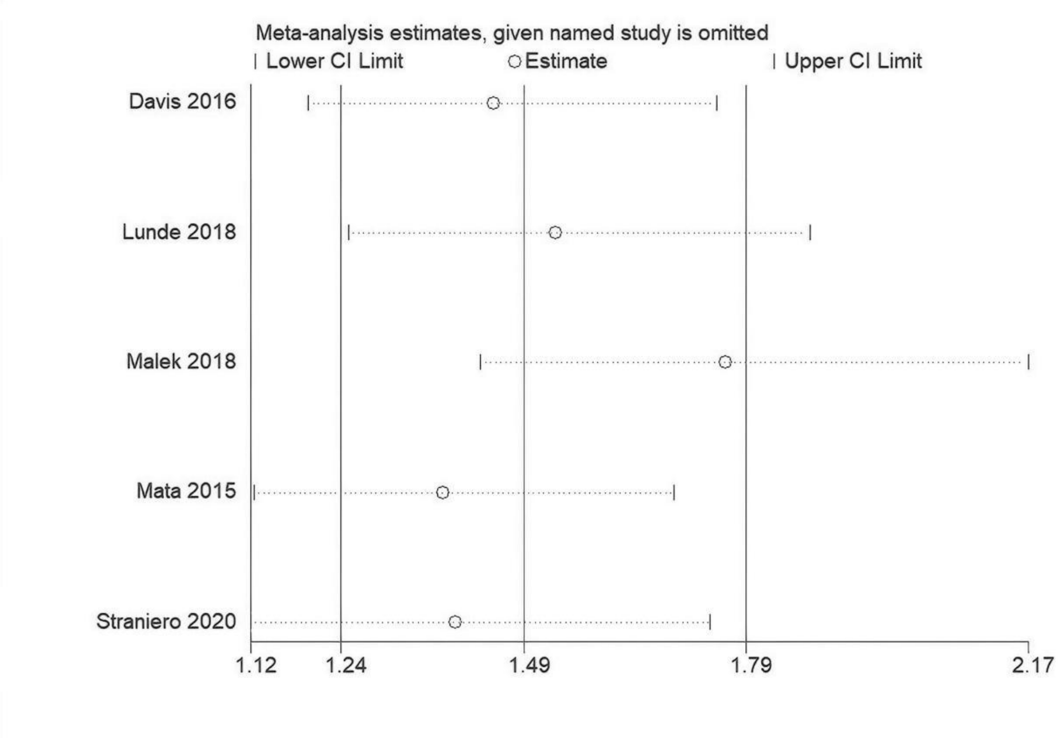

Supplement Figure 12. Sensitivity analysis for the association of N370S mutation with dementia risk

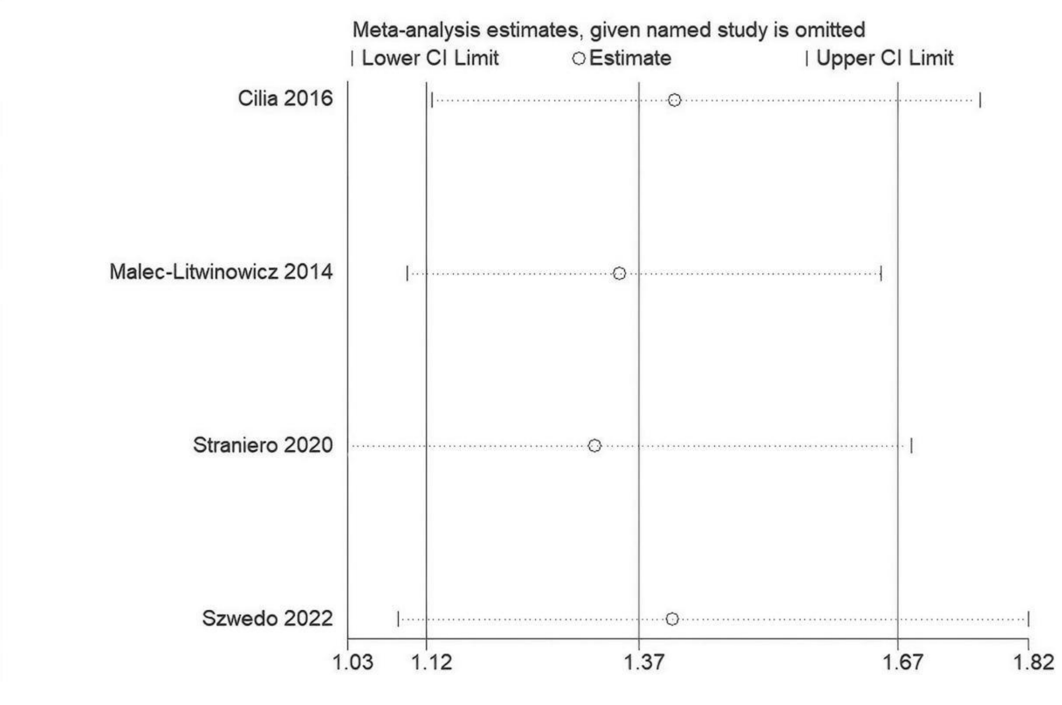

Supplement Figure 13. Sensitivity analysis for the association of L444P mutation with dementia risk

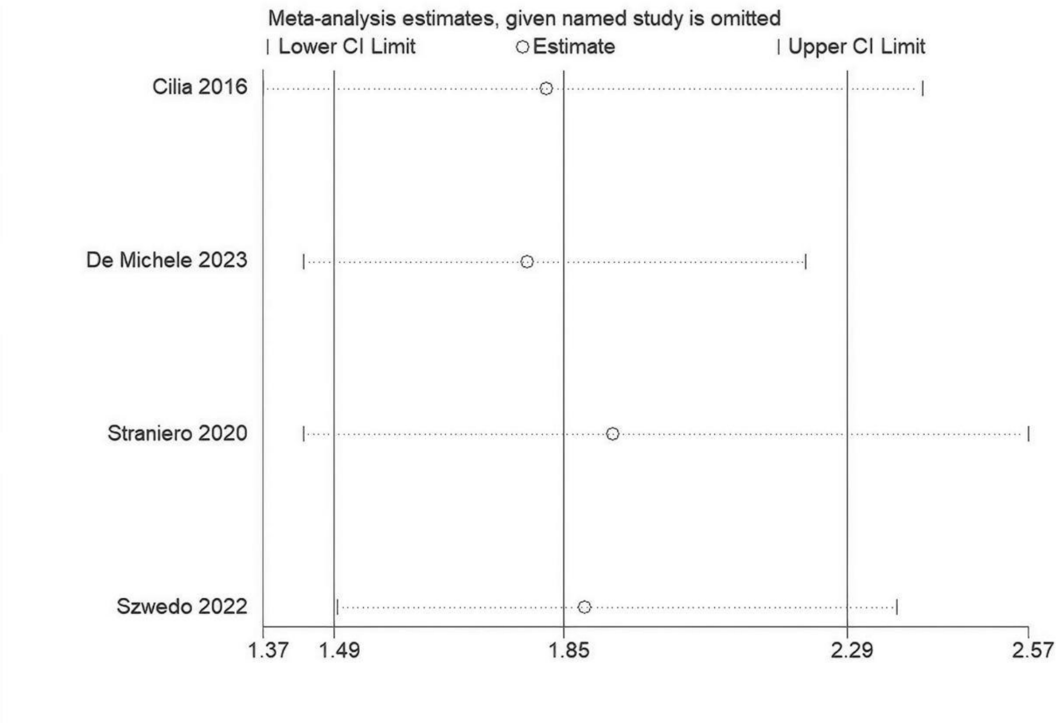

Supplement Figure 14. Sensitivity analysis for the association of E326K polymorphism with dementia risk

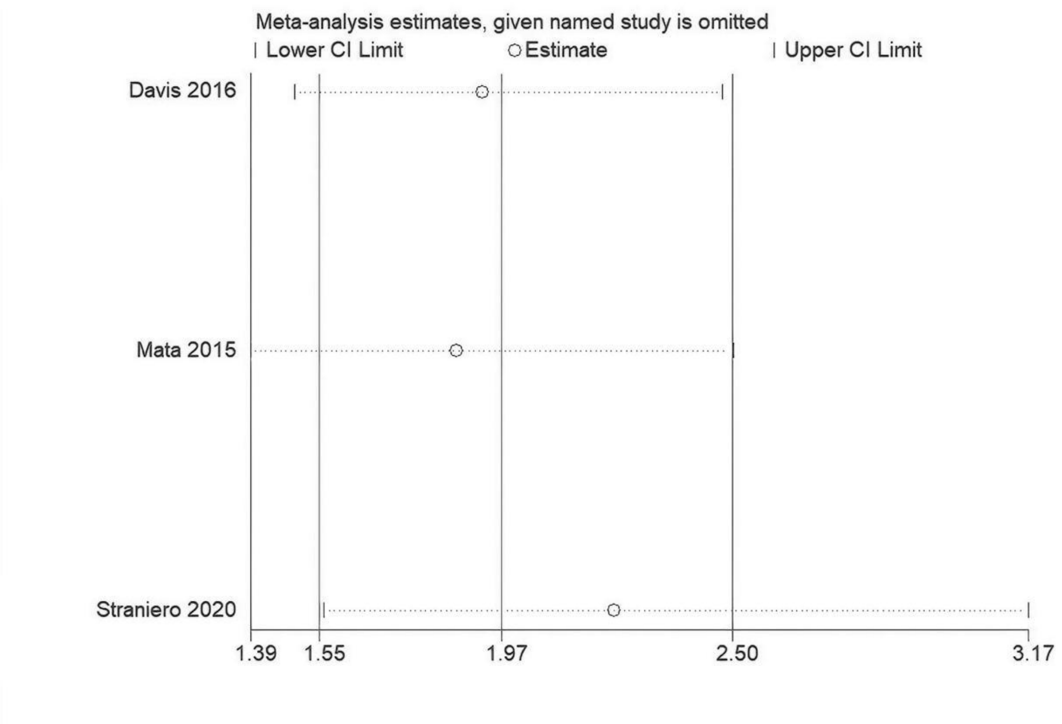

Supplement Figure 15. Funnel plot for the overall association of GBA variations with dementia risk

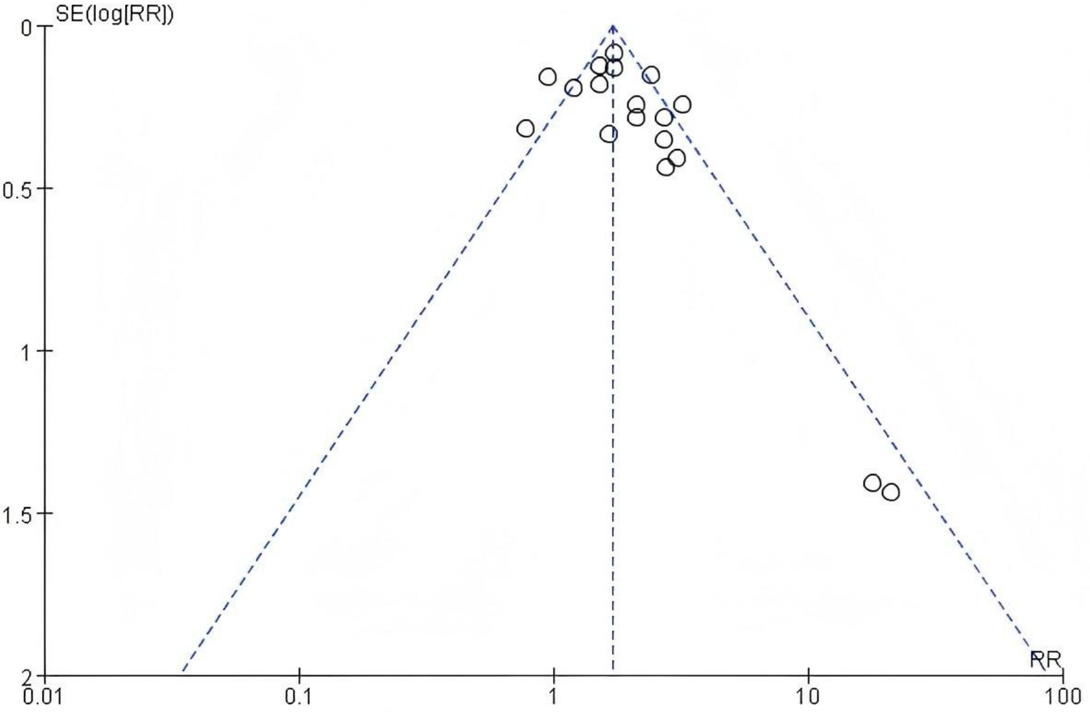

Supplement Figure 16. Funnel plot for the association of GBA mutations with dementia risk

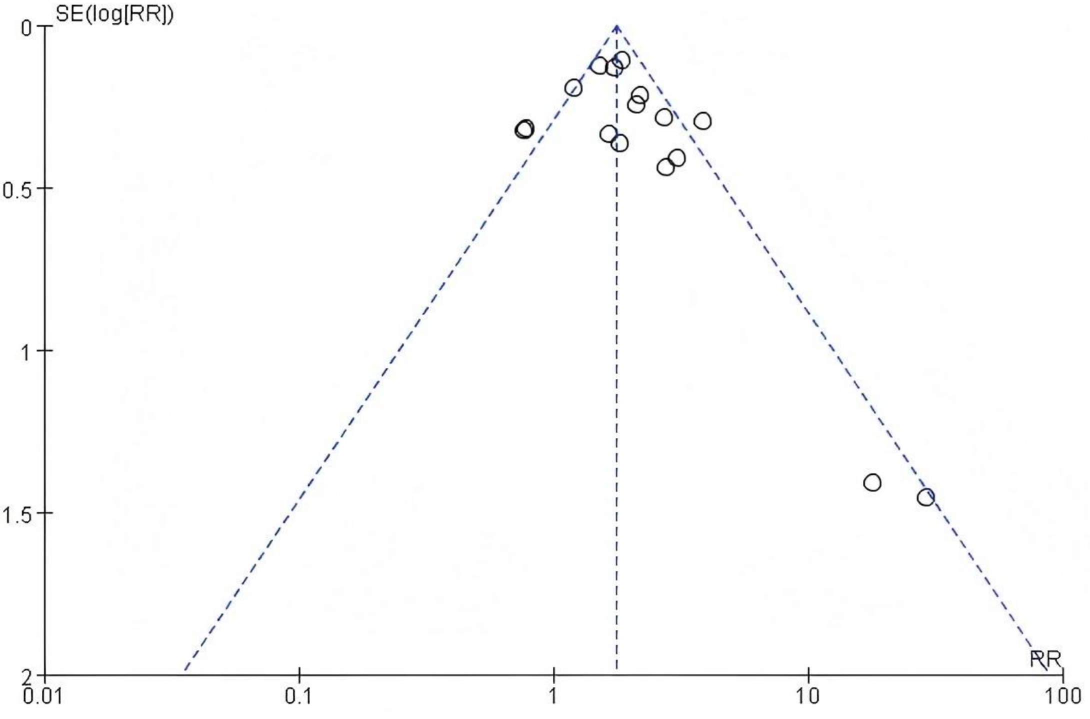

Supplement Figure 17. Funnel plot for association of GBA polymorphisms with dementia risk

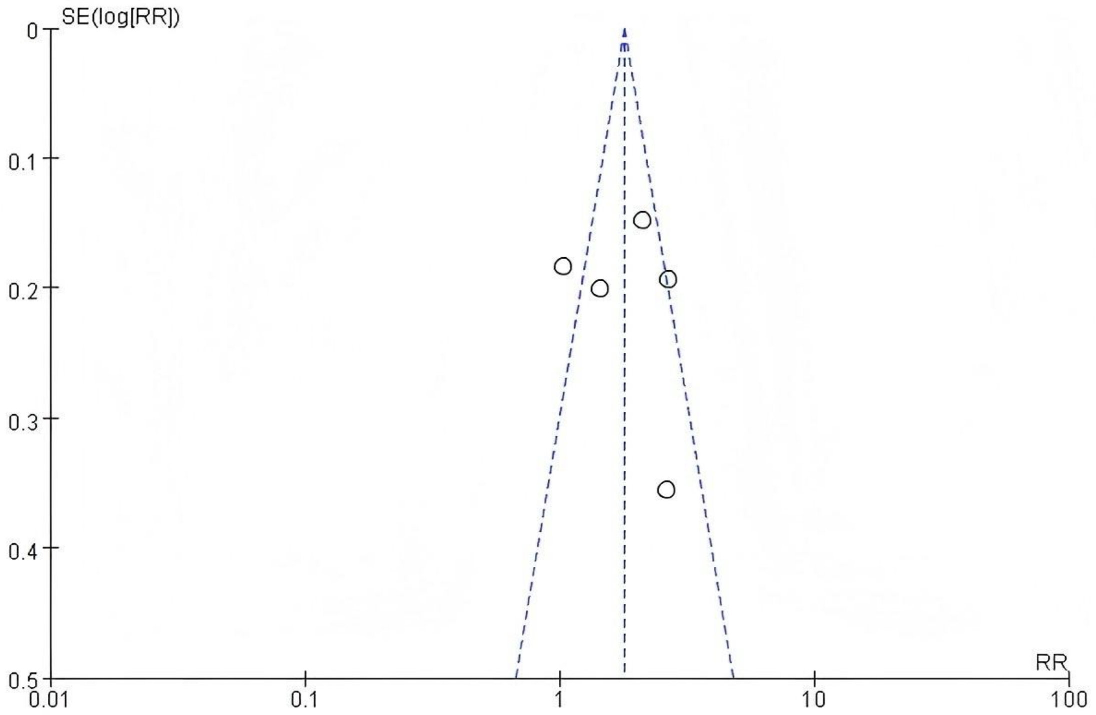

Supplement Figure 18. Funnel plot for the association of N370S mutation with dementia risk

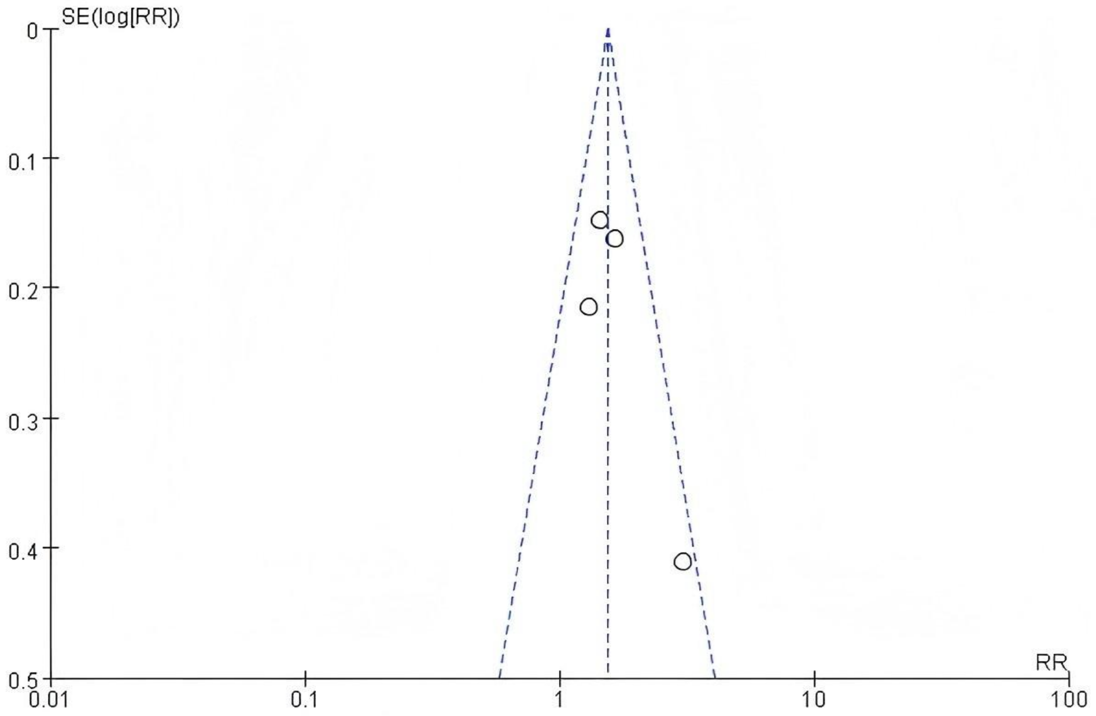

Supplement Figure 19. Funnel plot for the association of L444P mutation with dementia risk

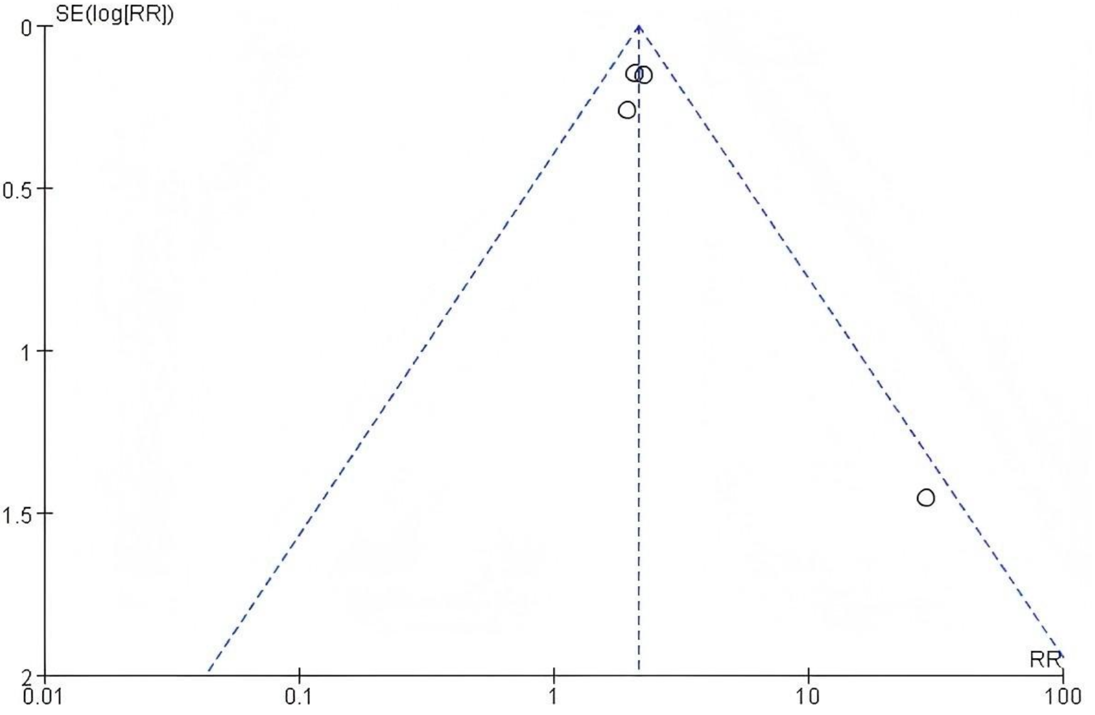

Supplement Figure 20. Funnel plot for the association of E326K polymorphism with dementia risk

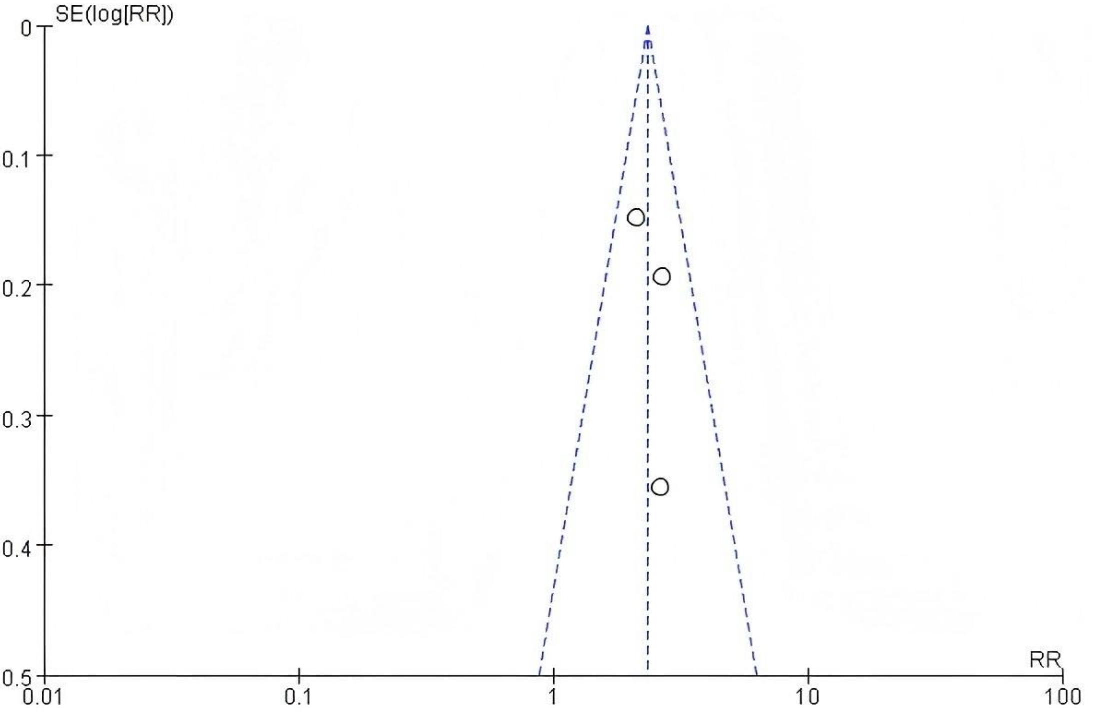

Supplement Figure 21. Egger's publication bias plot for N370S mutation

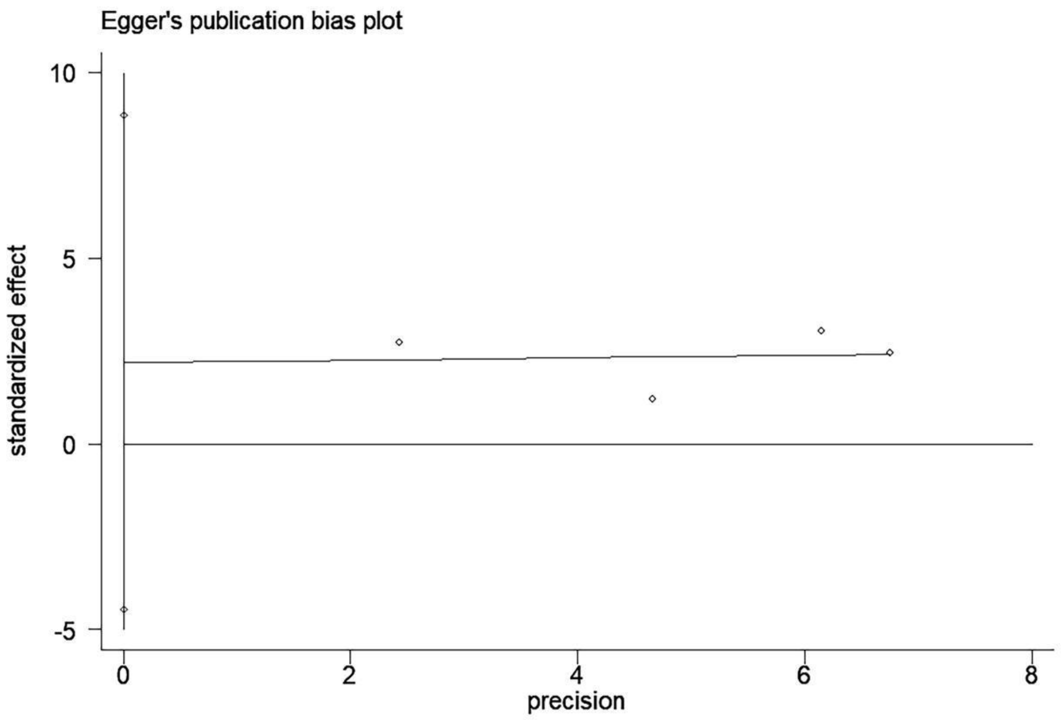

Supplement Figure 22. Egger's publication bias plot for L444P mutation

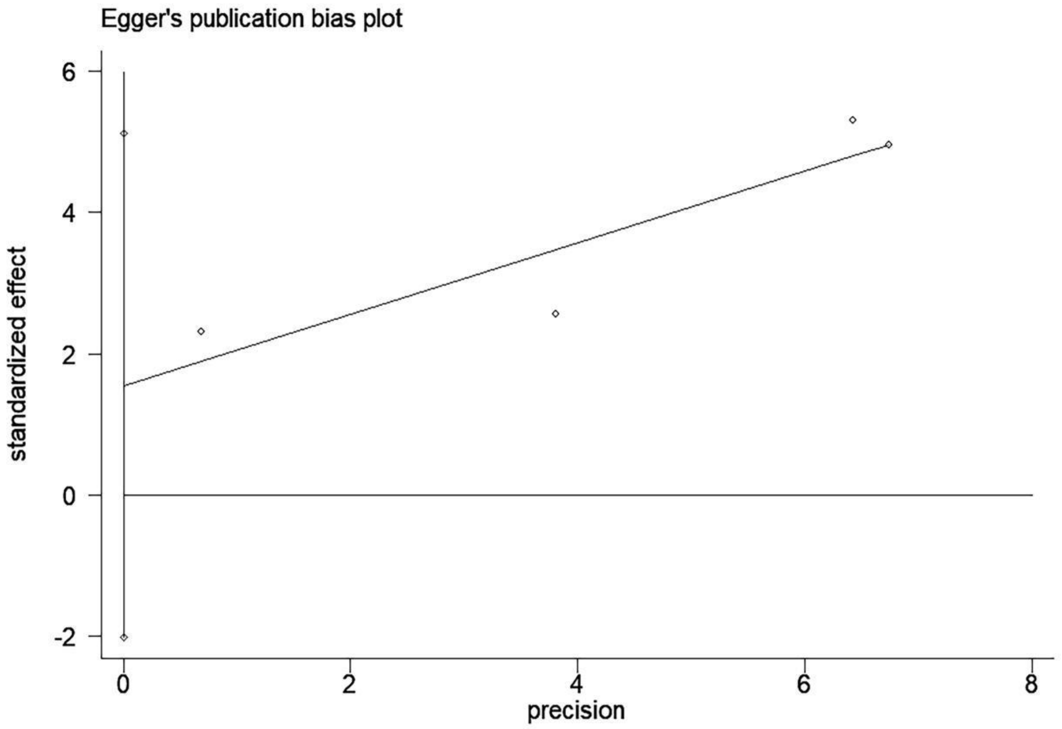

Supplement Figure 23. Egger's publication bias plot for E326K polymorphism

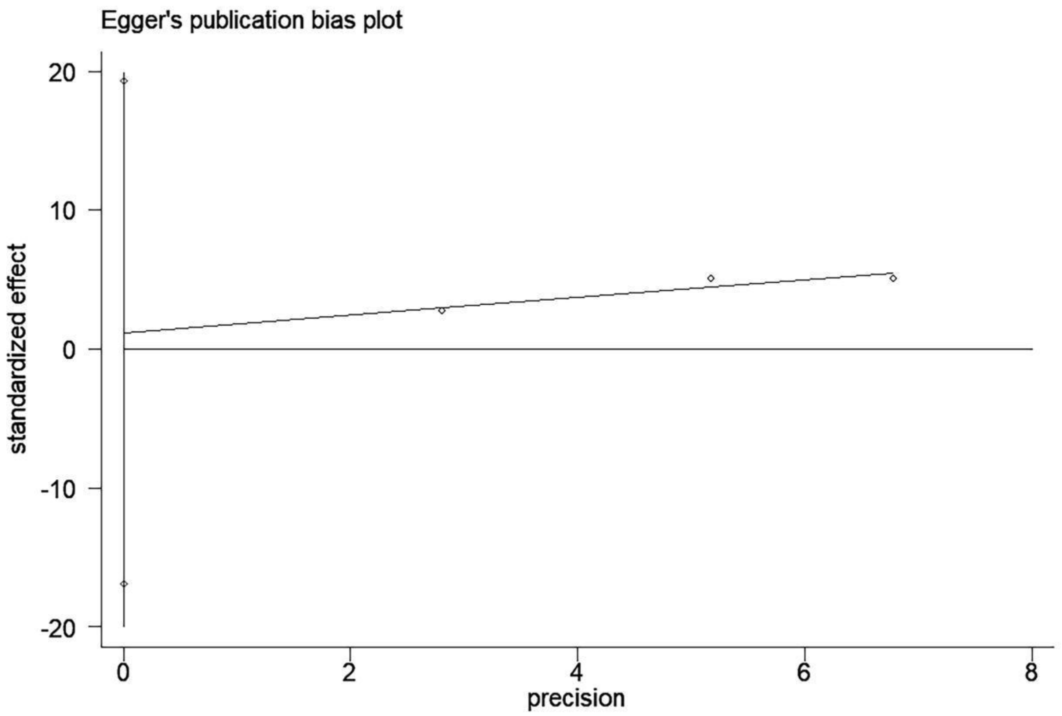

Supplement Figure 24. Egger's publication bias plot for GBA polymorphisms

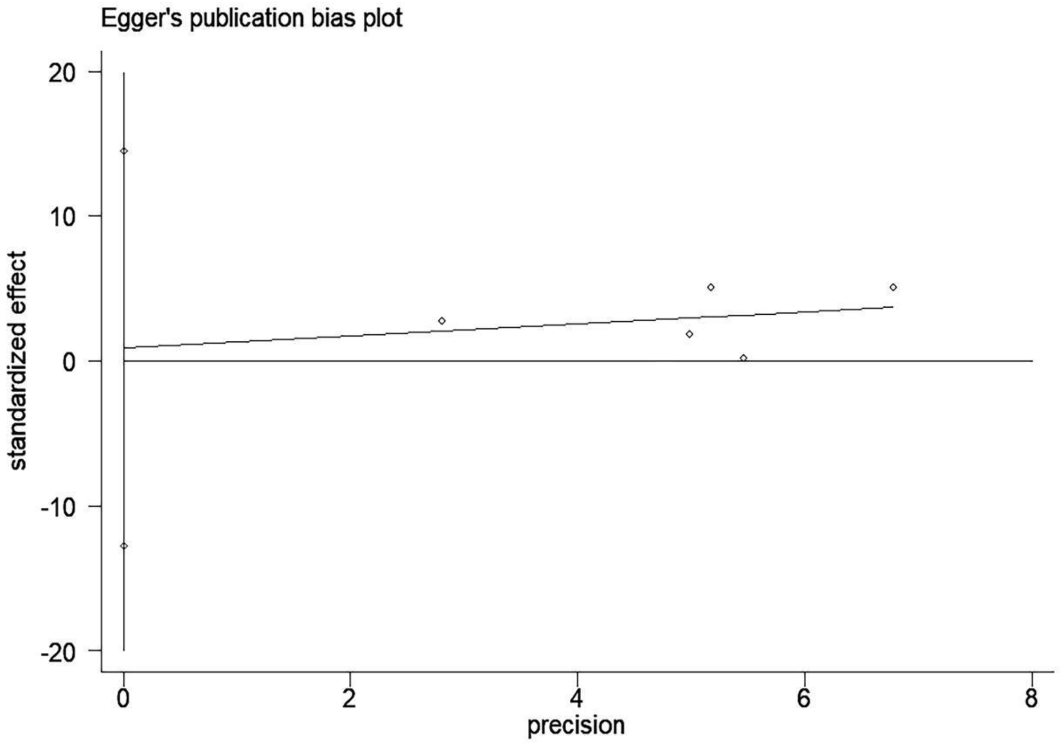

Supplement Figure 25. Egger’s publication bias plot for GBA mutations

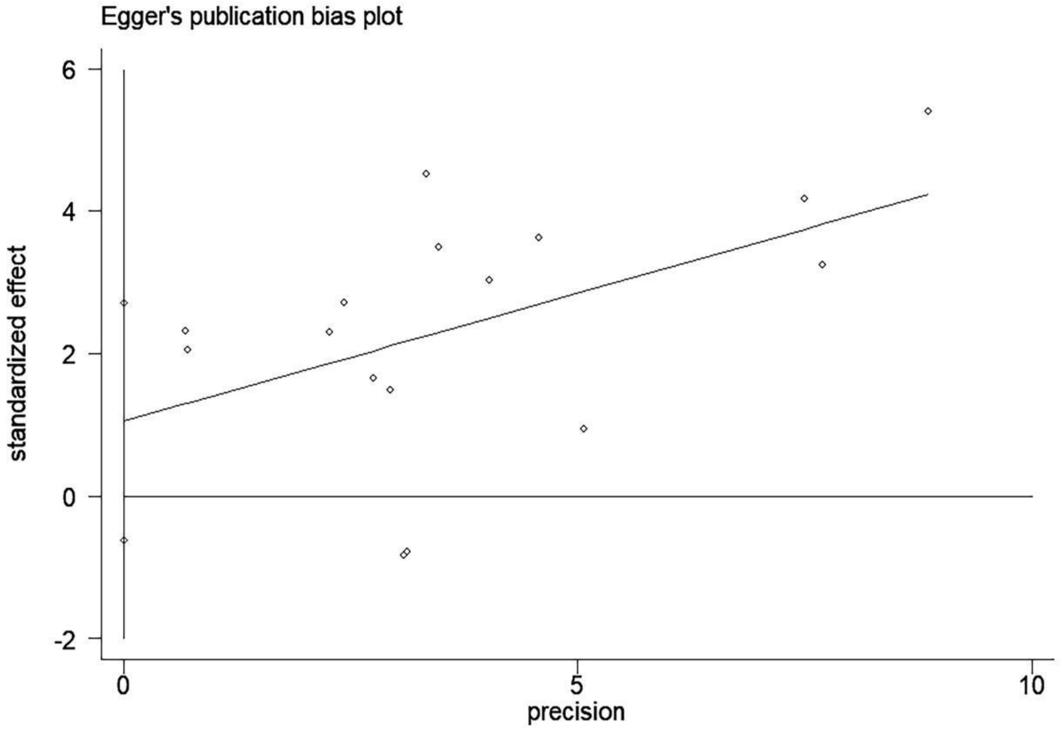

Supplement Figure 26. Egger’s publication bias plot for overall GBA variations

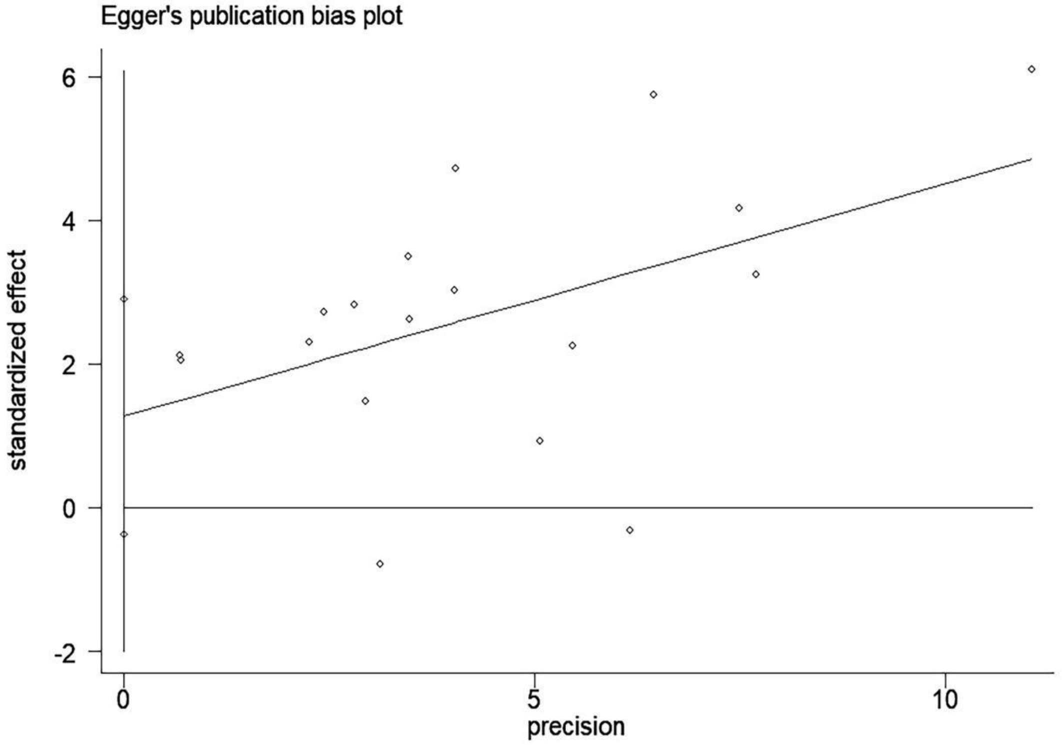

Supplement: Supplementary file 2 [file Data_Sheet_2.pdf]
